# Fiber optical parametric amplifiers in optical communication systems

**DOI:** 10.1002/lpor.201400087

**Published:** 2014-09-30

**Authors:** Michel E Marhic (†), Peter A Andrekson, Periklis Petropoulos, Stojan Radic, Christophe Peucheret, Mahmoud Jazayerifar

**Affiliations:** 1 College of Engineering, Swansea UniversitySwansea, Wales, UK; 2 Department of Microtechnology and Nanoscience, Chalmers University of TechnologyGothenburg, Sweden; 3 Optoelectronics Research Centre, University of SouthamptonSouthampton, SO17 1BJ, UK; 4 Department of Electrical and Computer Engineering, Jacobs School of Engineering, University of California San Diego9500 Gilman Dr, La Jolla, CA, 92093-0407, USA; 5 FOTON Laboratory, CNRS UMR 6082, ENSSAT, University of Rennes 1Lannion, France; 6 Department of Photonics Engineering, Technical University of Denmark, KgsLyngby, Denmark; 7 Technische Universität Berlin, Fachgebiet Hochfrequenztechnik-Photonics10587, Berlin, Germany; 8 In memory of Professor Michel E. Marhic, a pioneer in the research of fiber optical parametric amplifiers, who passed away unexpectedly during the preparation of this paper

**Keywords:** parametric amplifiers, fiber optic communication, fiber nonlinearities, phase-sensitive amplification, signal regeneration, phase regeneration, mid-span spectral inversion

## Abstract

The prospects for using fiber optical parametric amplifiers (OPAs) in optical communication systems are reviewed. Phase-insensitive amplifiers (PIAs) and phase-sensitive amplifiers (PSAs) are considered. Low-penalty amplification at/or near 1 Tb/s has been achieved, for both wavelength- and time-division multiplexed formats. High-quality mid-span spectral inversion has been demonstrated at 0.64 Tb/s, avoiding electronic dispersion compensation. All-optical amplitude regeneration of amplitude-modulated signals has been performed, while PSAs have been used to demonstrate phase regeneration of phase-modulated signals. A PSA with 1.1-dB noise figure has been demonstrated, and preliminary wavelength-division multiplexing experiments have been performed with PSAs. 512 Gb/s have been transmitted over 6,000 km by periodic phase conjugation. Simulations indicate that PIAs could reach data rate x reach products in excess of 14,000 Tb/s × km in realistic wavelength-division multiplexed long-haul networks. Technical challenges remaining to be addressed in order for fiber OPAs to become useful for long-haul communication networks are discussed.

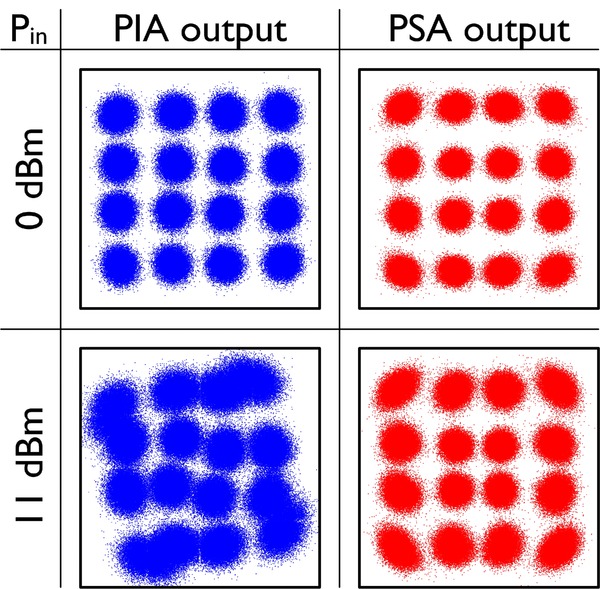

## 1. Introduction

In 1975 Stolen reported the first demonstration of optical parametric amplification (OPA) in low-loss optical fibers [1]. The introduction of erbium-doped fiber amplifiers (EDFAs) in optical communication systems in the late 1980s sparked interest in the possible development of fiber OPAs for communication systems. Fiber OPA research was greatly facilitated by the development of dispersion-shifted fiber (DSF) with zero-dispersion wavelength (ZDW) in the C-band around 1550 nm. This made it possible to use various fiber components being developed for communication systems. In 1995 DSF with nonlinearity increased by about a factor of 10 was developed [2], by reducing the core diameter and increasing its germania concentration. Because of their high figure of merit (ratio of nonlinearity coefficient *γ* to attenuation coefficient *α*), these highly-nonlinear DSFs (HNL-DSFs, or simply HNLFs) have since then remained the preferred medium for performing communication-oriented experimental work with fiber OPAs. In fact all the experiments reported in this review are based on such fibers.

Today EDFAs and distributed Raman amplifiers (DRAs) remain the two families of optical amplifiers deployed in communication systems. Hence this is an appropriate time to survey the state-of-the-art of recent research on fiber OPAs for communication systems, to assess their prospects for eventual penetration into future communication systems.

As their name indicates, fiber OPAs can in principle amplify optical communication signals. Just as EDFAs and DRAs, they can handle any modulation format, from binary modulation (on-off keying and binary phase-shift keying) to high-order amplitude and phase modulation formats, such as quadrature amplitude modulation (QAM), and amplify wavelength division multiplexed (WDM) signals. While the simplest OPA designs are polarization-sensitive, they can be modified to become polarization-insensitive. When operated as phase-insensitive amplifiers (PIAs), they can have noise figures (NF) of the order of 3 dB, similar to those of the current amplifiers. So in principle fiber OPAs can perform the same functions as these amplifiers. However, eventual substitution of fiber OPA for these amplifiers would require that they become competitive on a cost basis.

But fiber OPAs offer more than the possibility of a straightforward replacement of existing fiber amplifiers. Because they are based on significantly different physical principles, they can exhibit some characteristics which exceed those of current amplifiers, and they can also perform useful functions, which are not currently available. Some of these functions present unique advantages that could potentially be exploited for boosting the capability of optical communication systems beyond their current limits. Specifically these distinguishing features are:

(a) Adjustable gain spectra. The shape of the optical gain spectrum is determined primarily by the fiber dispersion properties, and it becomes wider as pump power is increased. Bandwidths of several hundreds of nanometers have been demonstrated, far exceeding the typical EDFA 35-nm bandwidth.
(b) Adjustable center frequency. Because the Kerr nonlinearity varies only slowly with wavelength, parametric gain can in principle be obtained around arbitrary pump wavelengths. This is only limited by the ability to design and manufacture fibers with corresponding dispersion properties.
(c) Wavelength conversion. Parametric amplification is based on four-wave mixing (FWM) associated with the third-order Kerr nonlinear susceptibility χ^(3)^ of fibers. FWM implies that if one or two high-power continuous-wave (CW) pumps are used, and a weak signal is introduced at the input, a new wave, the idler, will grow together with the signal within the OPA. This idler will carry the same information as the signal, and will therefore constitute a duplicate, but at a different frequency. This clearly has potential applications in wavelength routing, etc.
(d) Phase conjugation. The idler also exhibits a reversal of the optical phase compared to the signal. This phenomenon can be exploited for compensating the effect of fiber dispersion in transmission fibers, as well as for mitigating some detrimental nonlinear effects occurring in such fibers.
(e) Pulsed operation for signal processing. The Kerr nonlinearity has a response time of just a few femtoseconds. Thus the OPA properties can be varied at very high rates, up to the teraherz range. Potential applications are switching, sampling, format conversion, short pulse generation, etc.
(f) 0-dB noise figure (NF). OPAs can be operated as phase-sensitive amplifiers (PSAs), which require that both signal and idler with equal amplitude be present at the input, with a specific phase relationship. In theory such devices can exhibit an NF approaching 0 dB, i.e. about 3 dB better than current amplifiers. When used for periodic amplification in transmission links consisting of lossy fibers, degenerate (non-degenerate) fiber OPAs can exhibit a 3 (6) dB advantage in system noise figure, compared to EDFA-based systems [3, 4], This remarkable improvement could be exploited for increasing the transmission range, to accommodate higher-order modulation formats, etc.

In this paper we review recent progress in the field of fiber OPAs, which could have a significant impact on future optical communication systems. In Section 2 we review the basic physical principles of fiber OPAs, and their unique attributes which distinguish them from EDFAs and DRAs. In Section 3 we present two signal processing applications: Time domain demultiplexing, and all-optical amplitude and phase regeneration. In Section 4 we describe the use of OPAs for broadband and phase-sensitive amplification. In Section 5 we discuss technical issues that limit the current performance of OPAs, and desirable developments for making further progress. We conclude in Section 6 by commenting on the progress achieved to date, and discussing prospects for a possible future role for OPAs in optical communication systems.

## 2. Review of fiber OPAs

**Figure 1:**
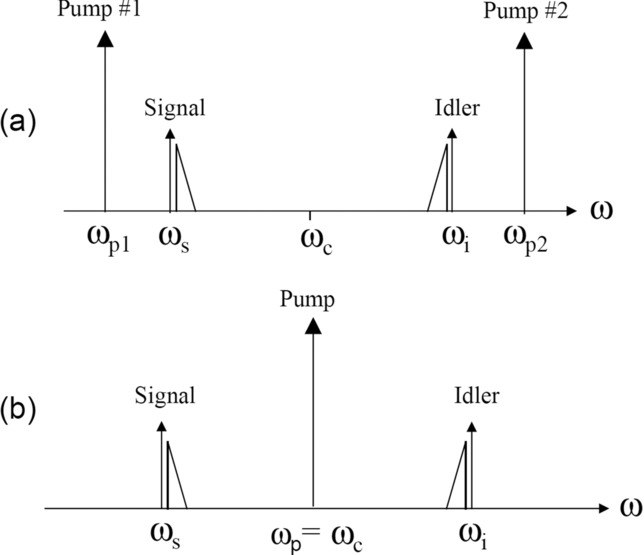
Sketches of the output spectra of fiber OPAs. (a) two-pump version; (b) one-pump version.

Fiber OPAs are based on the third-order Kerr nonlinearity of optical fibers. The fiber nonlinearity coefficient *γ* is of the order of 10 W^−1^km^−1^ for silica-based HNLFs. One or two high-power waves at the angular frequencies *ω*_p1_ and *ω*_p2_ serve as pumps. If a weak signal at *ω*_s_ is injected at the input, co-propagating with the pumps, it generates a new wave at *ω*_i_ which grows along with it. The idler spectrum is a mirror image of that of the signal with respect to the center frequency *ω*_c_ = (*ω*_p1_+*ω*_p2_)/2 (which is halfway between the pumps); this means that *ω*_s_+*ω*_i_ = 2*ω*_c_. The spectrum of the idler is inverted, and it is complex-conjugated. This is illustrated in Figs. 1(a) and 1(b) which show the two- and one-pump cases, respectively. The signal consists of a carrier represented by an arrow, and a single-sideband modulation spectrum represented by a triangle on the high-frequency side. The spectral shape of the idler is a faithful replica of that of the signal, with the addition of a mirror reflection, which corresponds to spectral inversion.

The most general mode of operation of an OPA is when both signal and idler are present at the input, possibly with different amplitudes. We let the indices *s*(*i*) denote the signal(idler). In a lossless fiber (power attenuation coefficient *α* = 0) the relationship between the two input fields 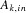 and output fields 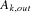, *k* = *s,i*, of such an OPA can be expressed as

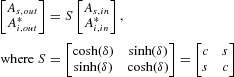
(1)
is the transfer matrix, and * represents complex conjugation. (To obtain this form it is necessary to introduce suitable phase shifts in the inputs and outputs.) *c* and *s* satisfy 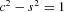 and 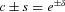. δ is given by

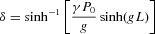
(2)

It is a function of the OPA parameters, which are: the fiber nonlinearity coefficient γ; the fiber length *L*; the total pump power *P*_0_ (in the case of two pumps, we assume that they have equal powers 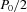); the small-signal parametric gain coefficient *g*, given by 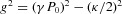; the total wavevector mismatch κ given by 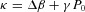 for two pumps and by 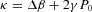 for one pump; 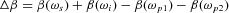 is the linear wavevector mismatch, which depends only on the fiber dispersion properties and the frequencies of the four waves; 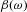 is the wavevector at the frequency ω.

When 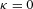, which corresponds to an ideal balance between linear and nonlinear phase-mismatch terms, the gain coefficient *g* has the maximal value 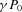, and *δ* has the maximal value 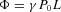. This is generally a desirable operating point, which can often be achieved in practice by choosing the frequencies so as to adjust 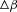. In optical communication we often seek to obtain nearly-maximum gain over as large a bandwidth as possible, and the design of suitable gain spectra is heavily influenced by the dispersion properties of the fiber used. In particular it is necessary to operate with 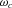 close to the zero-dispersion wavelength (ZDW), where the chromatic dispersion *D* vanishes, i.e. to have 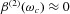, where 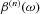 denotes the *n*^th^ derivative of 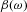. Then the fourth-order dispersion coefficient 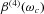 determines how wide the gain spectrum can be. It can be shown that for a well-optimized design, the bandwidth scales like 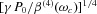
[5]. This shows the importance of having a fiber with a low 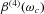 and a large *γ*. We also see that using a high pump power will increase the bandwidth. Experiments with CW pumps in the C-band routinely lead to bandwidths of several tens of nanometers, and several hundred nanometers have also been demonstrated with low-β^(4)^ fibers.

Most fiber OPA work uses one of two following main modes of operation:

(i) **Phase-insensitive amplifier (PIA)**.
  In this case the only waves injected at the input are the pump(s) and the signal. The only input electromagnetic field at the idler frequency is that due to vacuum fluctuations, which contribute to OPA noise. In this case it can be shown that the OPA gain is independent of the relative phase of the signal with respect to the pump(s). For this reason, this is referred to as a phase-insensitive amplifier (PIA). In this mode, a parametric PIA operates very much like an EDFA (which is of course phase-insensitive).
  For a well-optimized PIA the signal power gain, defined as the ratio of signal output power to signal input power, is equal to 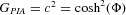, and when it is large its value in decibels is well approximated by 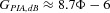. It is a fairly simple matter to obtain experimentally gains of tens of dBs. For example, if 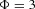, the maximum gain is approximately 20 dB; this could be achieved with a 1-km long DSF with 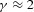 W^−1^ km^−1^, and a 1-W pump, or equivalently with a 100-m long HNL-DSF with 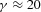 W^−1^ km^−1^, and the same pump power. The record gain for one-pump CW operation is 70 dB [6].
  We can calculate the NF of a PIA in a semi-classical manner, as follows [7]. We assume that the input signal is in a coherent state (CS), while the idler is in a vacuum state (VS). A property of CSs is that they have exactly the same level of quantum fluctuations as a VS (this is because a VS is a CS with zero mean field). If the input VS did not contribute to the output signal noise, the optical signal-to-noise ratio (OSNR) of the signal would not be degraded and the NF would be 0 dB. However, the input VS introduces the same level of noise at the output as the signal itself, and therefore the NF of the PIA is multiplied by 2 (3 dB). We note that this is the same as for an EDFA. (Of course in practice NFs are generally somewhat higher due to inevitable losses and other practical considerations.)
(ii) **Phase-sensitive amplifier (PSA)**.
  In this case the conditions at the input are the same as for PIA, with the crucial difference that now an additional finite-amplitude wave is introduced at the idler frequency. In general, signal and idler input amplitudes are about the same. The name ‘phase-sensitive amplifier’ means that in this case the gain depends on the relative phases of the waves. Specifically, if one performs the detailed calculation of the signal gain, keeping explicitly all the phase terms which were set aside to arrive at Eq. (1), one finds that this gain for a one-pump OPA depends on 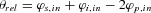, where 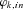 is the initial phase of the *k*th wave, *k* = *s,i,p* (see Section 4.3). Hence we see that a proper relationship must exist between the input phases in order to obtain and maintain a desired value for the gain. In practice this implies that PSAs need to incorporate some means for controlling this phase relationship, such as phase-lock loops, phase modulators, etc.
  We can also use Eq. (1) to find which combinations of signal and idler input phasors lead to the largest and the lowest gain. These combinations are the eigenvectors of *S*, and their gains are the corresponding eigenvalues. It can be shown that the eigenvectors have the form 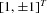 with the associated eigenvalues 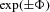. We note that in both cases signal and idler have the same amplitude, but that the phase difference between them is 0 for high gain and π for low gain. It should also be remembered that one of the inputs should be phase-conjugated (see Eq. (1)). In practice this means that the idler will have to be a phase-conjugated version of an initial signal. One way to accomplish this is to launch a signal into a PIA; then at its output both the signal and a conjugated idler will appear, as desired for presentation at the input of a PSA.
  The power gain for the high-gain eigenvector, defined as the ratio of signal output power to signal input power, is then 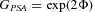. Its value in decibels is approximately 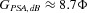, which is 6 dB larger than the gain of the same amplifier operated in the phase-insensitive mode (simply by turning off the input idler). This increased gain is one advantage of PSA vs PIA operation.
  The low-gain eigenvector, for which the input signal and idler are *π* out of phase (opposite signs), has the eigenvalue 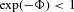. Hence the signal amplitude is actually attenuated rather than amplified. This is sometimes referred to as de-amplification. Clearly this mode of operation is not attractive for typical optical communication systems, where high gains are required. However, it has an important physical consequence, which could prove useful in sophisticated measurement systems. This is the fact that the de-amplification acts on quantum noise fluctuations (with the appropriate phase) as well as on deterministic classical signals. In particular it can be shown that by this means it is possible to reduce the noise power due to vacuum fluctuations, always present at the input of amplifiers, by 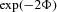. Any such reduction below the vacuum level is referred to in quantum optics as ‘squeezing’, and squeezed light has received considerable attention in the research literature. In classical communication systems, de-amplification or squeezing of unwanted classical fluctuations has found an application in the phase regeneration of phase-modulated signals. This aspect will be described in detail in Section 3.2.
  Interested readers can find in-depth treatments of the various concepts presented here in Ref. [8].

## 3. Optical processing for communication

In Section 2 we have described the operation of OPAs in the linear regime, which is suitable for amplifying communication signals with low distortion, as with EDFAs, Raman amplifiers, etc. However, different regimes of operation are also possible, which enable other functions which can also be used for manipulating communication systems in other ways. Such functions fall under the broad heading of optical processing. In this section we consider two such functions, namely demodulation of time-division-multiplexed (TDM) signals, and signal regeneration.

TDM demodulation is based on the use of a pulsed pump, which essentially turns an OPA into a high-speed switch. Since the response time of the optical Kerr effect in fibers is of the order of femtoseconds, such a switch can be used for picking out selected pulses with picosecond duration from a terabit/second stream of TDM pulses.

By contrast signal regeneration can make use of CW pumps, and it can in principle be used for regenerating phase and/or amplitude of certain types of signals with arbitrary symbol rates. Phase regeneration can be classified as a linear process, and can be understood on the basis of the theory presented in Section 2. On the other hand amplitude regeneration is based on gain saturation, which is not treated in Section 2 due to lack of space.

### 3.1. TDM demodulation

Parametric processes in *χ*^(3)^ waveguides are phase-preserving, femtosecond-scale processes [9] that allow for time-domain processing of signals with nearly-arbitrary bandwidths. In contrast to semiconductor materials with freely-generated carriers [10, 11], silica is characterized by a pure Kerr nonlinearity that leads to fast and efficient four-photon exchange by FWM in phase-matched mixers. Recognizing this advantage, Andrekson [12] pioneered signal sampling that relies on temporal gating defined by the pump duration and parametric mixer transfer function [8]. This original sampling concept can be generalized by combining signal spectral replication and subsequent sampling [13] of all newly-generated copies.

Before we describe such copy-and-sample preprocessor that can be used for time-division-multiplexing (TDM) of a very fast channel, it is instructive to consider the signal replication function first. (Also accomplished by FWM.)

**Figure 2:**
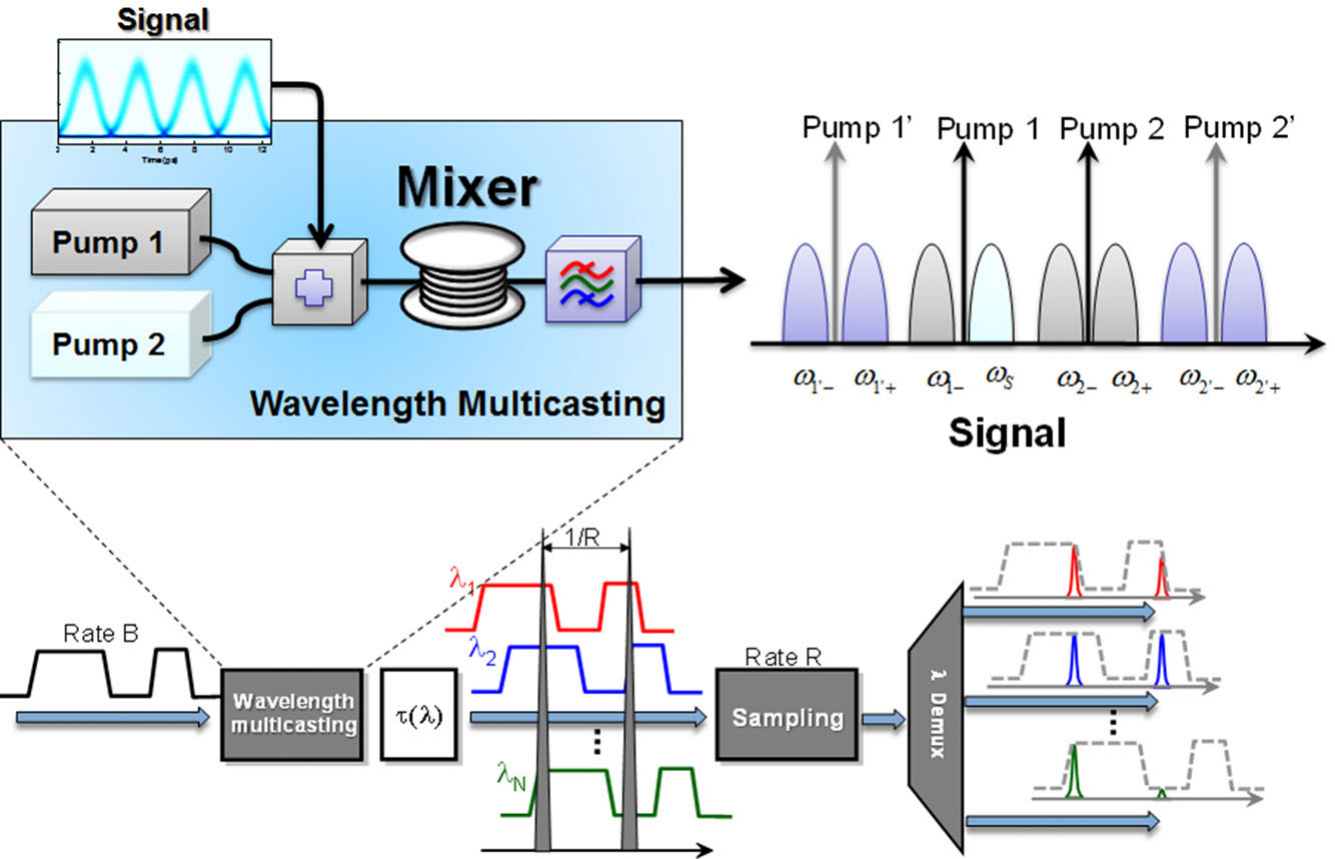
Top: Self-seeded parametric multicasting uses two CW pumps to simultaneously generate higher-order pumps (Pump 1′ and 2′) and create two channel copies with each pump wave. Bottom: Input signal at rate *B* is multicast to *N* spectral copies in parametric multicasting block; signal copies are mutually delayed in synchronization block (*τ*) and simultaneously sampled in parametric sampling block operating at rate *R*. The aggregate input rate is scaled to *N* subrate outputs at rate *B* = *N* × *R*.

Figure 2 illustrates the simplest principle for wavelength multicasting, that can be naturally scaled to a very large channel count [14]. Indeed, as long as the bandwidth of the multicasting mixer is sufficient to support generation of higher-order pumps (Pumps 1’ and 2’ in Fig. 2), it is possible to generate four new signal copies for each new pump order. Relying on such multicasting, Kuo et al. [14] recently generated more than 60 copies of a 10 Gb/s input channel.

**Figure 3:**
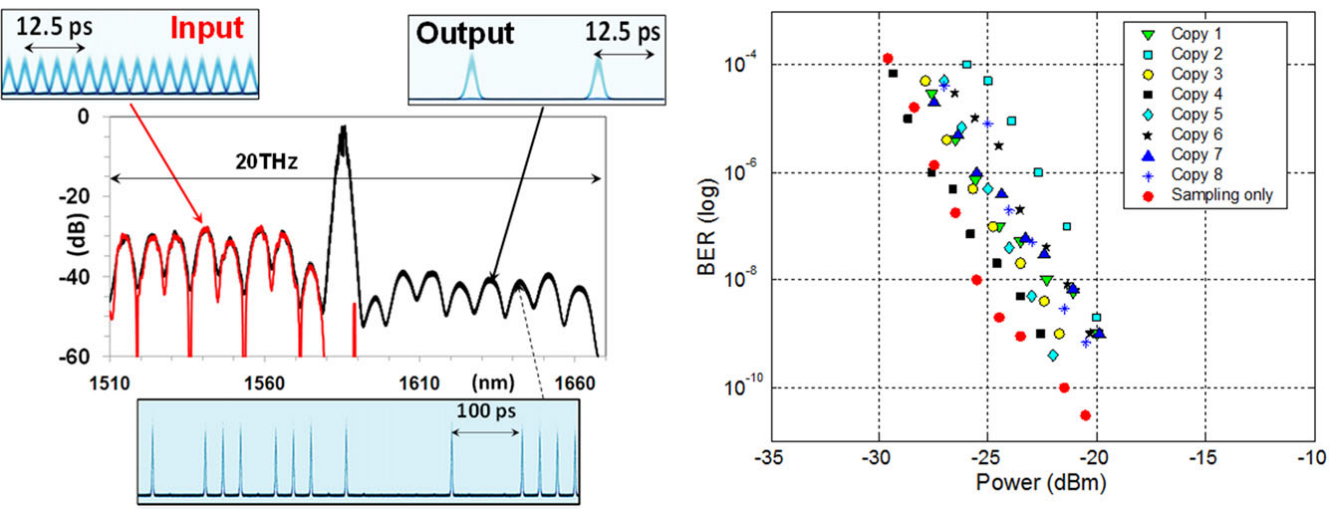
Optical spectra at the output of preprocessor (left). BER measurements as function of power on all 8 sampled copies compared to single wavelength sampling operation (right) [13].

The multicasting mixer can be followed by a single sampling gate to create a real-time TDM processor that can operate (sample) at much lower speed than the input signal rate (Section 7.5.1 in [8]) as shown in Fig. 2. While a multicasting subsystem can be realized using a CW-pumped mixer, the sampling block represents a true polychromatic sampling gate. In the case when input signal is digital, the processing chain can afford significant distortions imposed by the nonlinear mixer transfer function [15]; this, however, is not the case when the copy-and-sample processor operates on an analog or multilevel signal [16]. In such a case, the basic design limitation is set by the bandwidth of both parametric mixers: generation of *N* spectral copies requires a CW mixer capable of power-equalized amplification (conversion) over a range that is sufficient to accommodate 2*N* copies of the input channel. In the case when the signal is a 320 Gb/s RZ-modulated channel, this means that both multicaster and sampler must possess 10 THz minimal bandwidth to generate 40 Gb/s subrate outputs. In practice, this bandwidth is almost doubled in order to accommodate spectral bandwidths necessary for filtering and pump-signal separation, as shown in Fig. 3.

**Figure 4:**
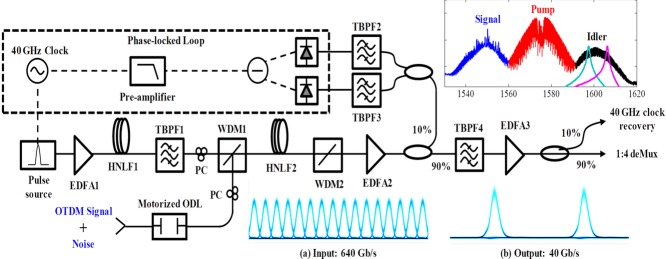
Self-tracked FPM-CPM gate. Inset shows the input 640 Gb/s and output 40 Gb/s eye diagrams, and the output spectrum of HNLF2. The transmission spectra of TBPF2 and TBPF3 are overlaid on the idler spectrum. Fast temporal variations (256 ps/s) resulted in 0.8 dB sensitivity penalty [16]. HNLF: Highly Nonlinear Fiber; EDFA: Erbium doped fiber amplifier; WDM: wavelength division multiplexer; TBPF: Tunable bandpass filter.

As the rate of the input signal grows, it is not sufficient to build a wideband mixer but is also necessary to maintain a precise timing and to synchronize the sampling gate operation with the input channel time reference. While it is possible, at least in principle, to envision an optical phase-locked loop (OPLL) to be used in this role, this approach inevitably leads to lower sensitivity. In contrast, a single parametric mixer can perform combined clock recovery and demultiplexing by relying on parasitic cross-phase modulation (CPM) induced between unsynchronized pump and signal. A self-tracked OTDM architecture is shown in Fig. 4, which automatically tracks clock in of a 640 Gb/s channel, even in the case when fast temporal drift is induced [17, 18].

### 3.2. Regeneration

In response to the ever-increasing demand for transmission capacity of the Internet backbone, new ways of encoding and transmitting information in fiber optic cables have been sought for. The most evident consequence of this over the last decade has been the widespread adoption of modulation formats that encode information in the phase (rather than the intensity) of the optical carrier. Apart from paving the path towards more spectrally efficient communications, encoding an optical signal in phase also has the advantage of offering resilience to certain linear and nonlinear transmission impairments [19]. On the other hand, the introduction of phase encoding gives rise to nonlinear phase noise, which manifests itself as a new dominant limitation to system performance [20–22]. In amplified transmission systems, nonlinear phase noise originates from the combined effects of noise introduced by the in-line optical amplifiers and the nonlinear interaction between the various co-propagating WDM channels. It is therefore necessary for signals transmitted over long distances to be regenerated periodically. This currently occurs in installed transmission systems by employing optical-to-electronic-to-optical (O/E/O) conversion and fast electronic processors. However, techniques that can perform regeneration by avoiding the need for O/E/O conversion merit attention, since they are often associated with additional attractive features, such as transparency to the symbol rate and a more favorable scaling of the energy consumption with higher repetition rates as compared to electronics.

In the past, some of the all-optical techniques that have been used for the mitigation of noise in phase-encoded signals have been based either on phase-preserving amplitude regeneration [23–26], or phase-to-amplitude format conversion and subsequent amplitude regeneration (before converting back to a phase-encoded signal if further transmission is required) [27]. Phase-preserving amplitude regenerators are useful for preventing nonlinear phase noise build up, suggesting their use as in-line regenerators. However, their ability to improve the BER when placed in front of a receiver is only limited, since they can remove amplitude but not any phase noise. Regeneration through phase-to-amplitude format conversion also suffers from the same issue, since it is equivalent to performing detection prior to regeneration [28]. In addition, because this scheme operates on the intensity rather than directly on the electric field of the demodulated signal, it may have a lower tolerance to phase noise.

**Figure 5:**
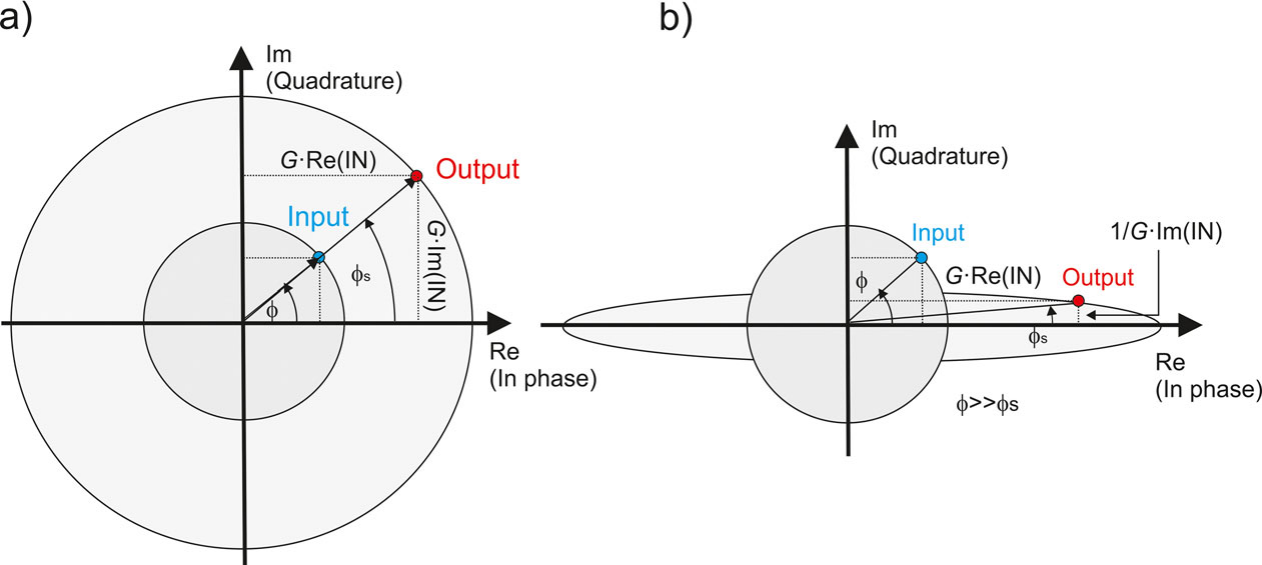
A comparison between phase-insensitive (a) and phase-sensitive amplification (b), as depicted in the complex plane.

Counter to the aforementioned approaches, an implementation based on phase-sensitive amplification alleviates these issues, since it operates directly on the phase. In order to understand how a PSA can regenerate the phase of the optical carrier, it is useful to refer to the diagrams presented in Fig. 5. In a PIA, such as an EDFA (Fig. 5a), the in-phase and quadrature signal components experience identical gain, and as a result the field amplitude is amplified with its phase unaffected. In a PSA on the other hand, the two quadrature components experience a different amount of gain (Fig. 5b). For example, in a PSA based on degenerate FWM [29], the in-phase component of the electric field experiences gain *G*, while the quadrature component is de-amplified by 1/*G*. Consequently, the output phase of the amplified signal is more closely aligned towards the amplifier's in-phase axis, as shown in Fig. 5b. This phase modification effect, known as ‘phase squeezing’, is inherently suitable for the regeneration of binary phase-encoded signals, as the ‘phase-squeezed’ data bits are forced to adopt a phase of either 0 or π, thus restoring the fidelity of the signal prior to transmission. However, Fig. 5b also shows that a side-effect of the PSA action is the introduction of unwanted amplitude variations; the larger the phase error the PSA corrects for, the larger the amplitude variations at its output. This can be avoided by operating the PSA in saturation. Then any amplitude noise present in the phase-encoded signal can also be reduced, thereby enabling simultaneous phase and amplitude regeneration.

We also note that when a two-pump PSA is driven into saturation, higher-order sidebands are generated by FWM, and these can be exploited to improve the extinction ratio of phase regeneration [30].

The implementation of a PSA based on parametric processes has its own challenges: In order to achieve phase-sensitive amplification, the phase relationship between the PSA pump(s), signal and any idlers present needs to be maintained, that is all the waves participating in the parametric process need to be phase-locked [31]. This is challenging in practice due to two main reasons: (i) phase-encoded signals often have the carrier field suppressed (i.e. they are ‘carrier-less’) and (ii) even if the carrier were extractable, it would generally contain a part of the phase and amplitude noise of the transmitted data. Proof-of-principle PSA demonstrations have overcome this problem by ensuring that both the signal and the pump(s) originate from a common laser source, e.g. through the generation of an optical frequency comb. This would not be possible in a system considered for a real transmission link, in which case any pumps would have to be generated locally in the regenerating node, i.e. they would have to be truly independent from the data signal.

An additional challenge, which is nevertheless common in several OPA configurations, is that strong CW signals need to be used as the PSA pumps. When the nonlinear element used for the implementation of the PSA is an optical fiber (which is arguably the most mature technology for the observation of this type of nonlinear effects today), a strong limitation to the amount of power that can be launched to the fiber is determined by the onset of any stimulated Brillouin scattering (SBS) effects. Typically, this issue is tackled by phase-dithering the CW pump with a frequency spectrum that is broader than the SBS gain bandwidth. However, this technique can obviously not be directly applied in a PSA, since it would disturb the phase relationship between the various waves.

In the remaining part of this section and through the description of the experimental demonstration of a PSA-based phase and amplitude regenerator for binary phase-shift keyed (BPSK) signals [28, 32], we will show how these challenges can be overcome to result in a regenerating system suitable for use in transmission links. Subsequently, we will show how this basic operating principle can be extended to accommodate processing of more complex modulation formats.

#### 3.2.1. A PSA-based BPSK phase and amplitude regenerator

**Figure 6:**
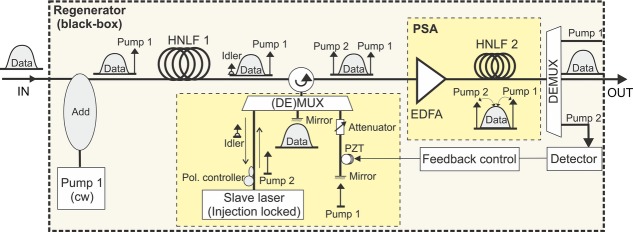
Experimental set-up of a PSA-based regenerator of BPSK signals ((DE)MUX: De/multiplexer; EDFA: Erbium-doped fiber amplifier.

**Figure 7:**
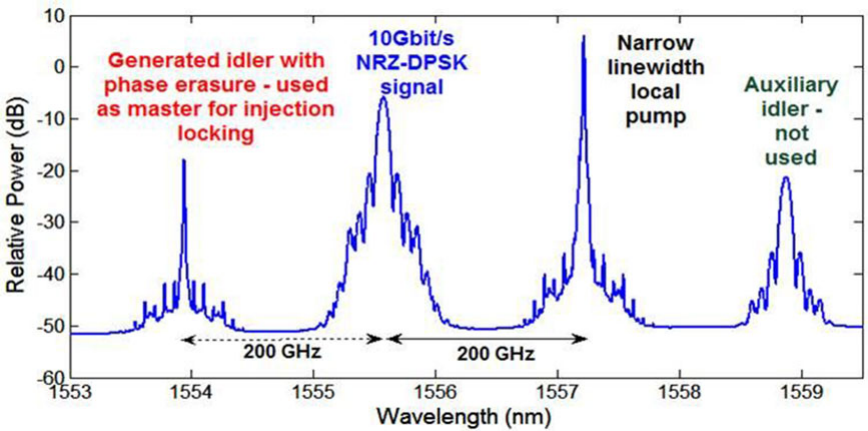
Spectral trace obtained at the output of HNLF1 showing the generation of the narrowband (BPSK data-free) idler.

The experimental set-up of a PSA used for the regeneration of a (remote) BPSK signal is shown in Fig. 6 [28]. The majority of the regenerator set-up used polarization-maintaining (PM) components, with the exception of the section marked as “Different paths box” and the nonlinear fiber HNLF2. At the input of the regenerator, the signal was combined with a free-running narrow-linewidth CW pump (Pump1) using a low-loss add/drop multiplexer. The pump operated 200 GHz away from the signal wavelength with a power of 17 dBm. The combination of pump and signal generated a FWM idler through propagation in a length of polarization-maintaining HNLF (HNLF1, *L* = 300 m, chromatic dispersion coefficient *D* = −0.01 ps/nm/km at 1550 nm, *γ* = 10.7 /W/km and 0.9 dB/km loss), as shown in Fig. 7. This spectral trace shows that the generated idler was narrowband, i.e. any data modulation was stripped off from it. This is a consequence of the relation between the idler phase 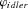 and the BPSK data signal phase 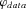, i.e. 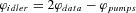, due to which any π phase jumps existing in the BPSK data are erased in the idler. An additional consequence of this relation is that the phases of the group of three waves (data, pump1 and idler) were now locked relative to each other, thus allowing their phase-sensitive interaction in a subsequent parametric amplifier.

Figure 6 shows the experimental configuration of a PSA used for the regeneration of a (remote) BPSK signal [28]. Operation of the system is based on the generation of a set of two pumps which together with the signal are mutually phase-locked. This is achieved by combining the data signal with one independent pump (Pump 1 in the figure) and mixing the two in an OPA (HNLF1), see Fig. 7. The phase of the resulting idler 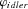 then relates to that of the two other waves as 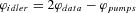. This relation shows that not only have these three waves a constant phase relation, thus allowing their phase-sensitive interaction in a subsequent parametric amplifier, but additionally, any π phase jumps existing in the BPSK data are erased in the idler, i.e. the data modulation was stripped off from it. This can be appreciated in Fig. 7 by noticing how narrowband the generated idler is with respect to the signal. Conversely however, any phase noise present in the data (i.e. any deviations from π in the data modulation) is transferred to the idler through this process, manifesting itself as spectral broadening. These undesired frequency components are eliminated in the following stage of the set-up: The three waves are separated in a de/multiplexer placed behind a circulator. Whilst the data and Pump 1 are simply retroreflected in a mirror and directed back into the multiplexer, the idler is used to injection-lock a discrete-mode semiconductor laser. Injection-locking acts as a narrowband filtering mechanism that rejects the noise present in the idler, while it effectively amplifies the power of the CW light. The bandwidth of the injection-locking process can be controlled through the injection power and is normally around a few hundred MHz. Therefore, even though the majority of high-frequency noise is rejected, some low-frequency noise content may still be partly transferred, potentially limiting the PSA regeneration performance at low frequencies.

Once the three waves are recombined at the output of the de/multiplexer, they constitute a two-pump arrangement for degenerately amplifying the data signal in the OPA formed in HNLF2. Moreover, because of the constant phase relation between the three waves, this OPA operates in a phase-sensitive fashion. An important advantage of this configuration is that all the optical waves involved in the process share a common path through most of the regenerator, except for the output side of the demultiplexer. The lengths of fiber at the demultiplexer output ports can be kept short and can all be in close proximity during packaging to ensure that they experience similar acoustic/thermal pick-up. This ensures that the phase-locking condition is maintained throughout and the environmental sensitivity of the system is minimal.

More details on the experiments that were conducted on this system can be found in Ref. [[28]]. An additional feature of these experiments included the use of a fiber with an alumino-silicate core and a linear strain gradient along its length (HNLF2), to ensure that relatively high pump powers could be used without suffering from the onset of SBS effects. Furthermore, in order to ensure regeneration of the signal amplitude as well as the phase, the PSA was operated in deep saturation. Monitoring the power of the depleted pumps provided a convenient means of providing feedback for the adjustment of the absolute phase of waves at the PSA input (see Fig. 6), so as to ensure that it operated at maximum PSA gain for the data.

**Figure 8:**
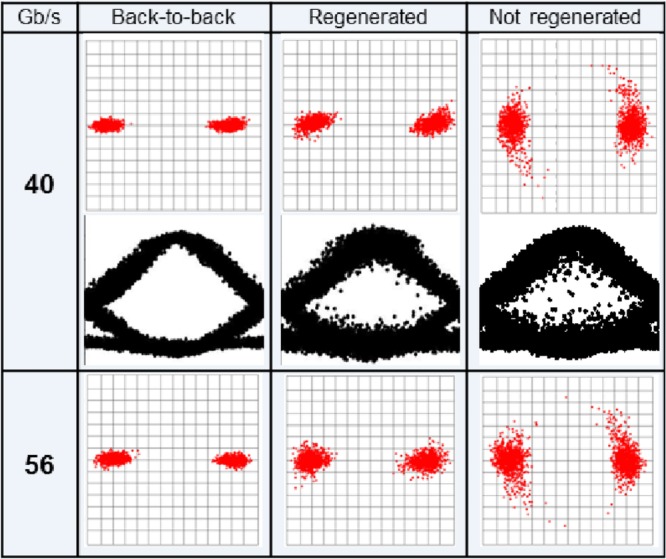
Constellation diagrams for back-to-back and for added phase noise for two data rates and the corresponding demodulated eyes for 40 Gb/s (see [28] for more details).

The response of the regenerator was initially tested in the laboratory with BPSK signals operating either at 40 or 56 Gb/s. To emulate nonlinear phase noise, the data signal was further modulated using a LiNbO_3_ phase modulator driven by white noise with a 3-dB bandwidth of 16 GHz. Relatively high levels of phase noise were introduced to the signal, in order to test the resilience of the regenerator. Figure 8 shows constellation diagrams and eye diagrams (at 40 Gbit/s only) of the original signal (with and without phase noise added) and the signal at the output of the regenerator. The results confirm that the phase noise can be significantly squeezed by the regenerator with negligible induced amplitude noise, and also illustrate the bit rate transparency of the scheme.

**Figure 9:**
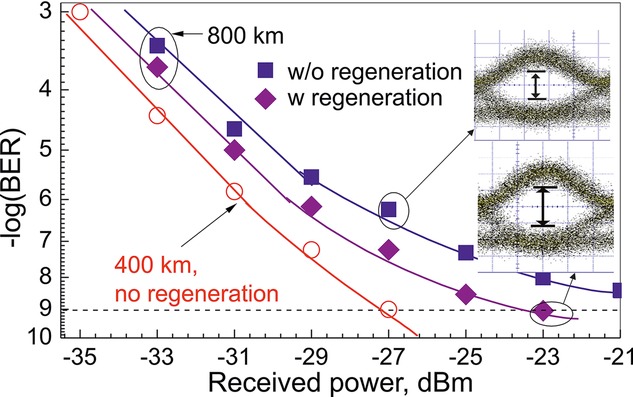
BER curves (and eye diagrams) of a DPSK signal after transmission over 400 km and 800 km of fiber, with and without PSA-based regeneration.

The regenerator was also tested in transmission using a section of the UK's Aurora dark fiber link [33]. For these tests, a 40 Gb/s differential phase-shift keying (DPSK) signal was coupled together with another 36 WDM channels carrying similar data and transmitted over 400 km in the installed fiber link. The signal was regenerated at that point before being re-transmitted over another 400-km length of the same fiber (in order to facilitate re-transmission of the data, the regenerator was set up to perform wavelength conversion as well as regeneration of the signal). A commercial polarization tracker was used at the input of the regenerator to ensure that the polarization of the signal remained aligned to that of Pump 1. Figure 9 presents a summary of the BER results obtained from these experiments. The figure shows that an error floor above a BER of 10^−9^ was observed when the regenerator was not in place. Use of the regenerator helped restore the quality of the signal and reduced the error floor to below this level.

#### 3.2.2. Extension to more complex phase-shift keying formats

We saw in the previous paragraphs how a FWM-based PSA can be made to operate as a two-level quantizer, restricting the phase of the optical carrier to just the values of 0 and π radians. Moreover, the overall discussion on OPAs presented so far has revealed that a whole host of functionalities that act on the phase of the optical carrier can be implemented. For example, by exploiting the basic phase relationship between the signal and the generated idler in a single-pump non-degenerate OPA (*φ_idler_* = 2*φ_pump_* – *φ_signal_*), we see that phase reversal (conjugation) can readily be achieved. In the same manner, if the idler is present at the input of the system, then coherent addition of the two waves is obtained at the output. Furthermore, by considering the pump wave as the reference in the same example, the same relationship shows that doubling of the pump phase is achieved in the OPA, whereas different multiplication factors can be obtained by generating higher-order mixing components. In the context of regeneration of an *M*-level phase modulated format, the aforementioned functionalities can be combined to give rise to a staircase phase response, which mathematically can be expressed as [32, 34]:

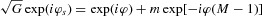
(3)
where *m* is a weighing factor, *φ* and *φ_s_* are the phases of the input and output signal respectively and *G* is an accompanying intensity modulation response. Following from Eq. (3), the intensity and phase of the signal at the output can be expressed as a function of the input phase as below:

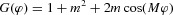
(4)

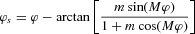
(5)

**Figure 10:**
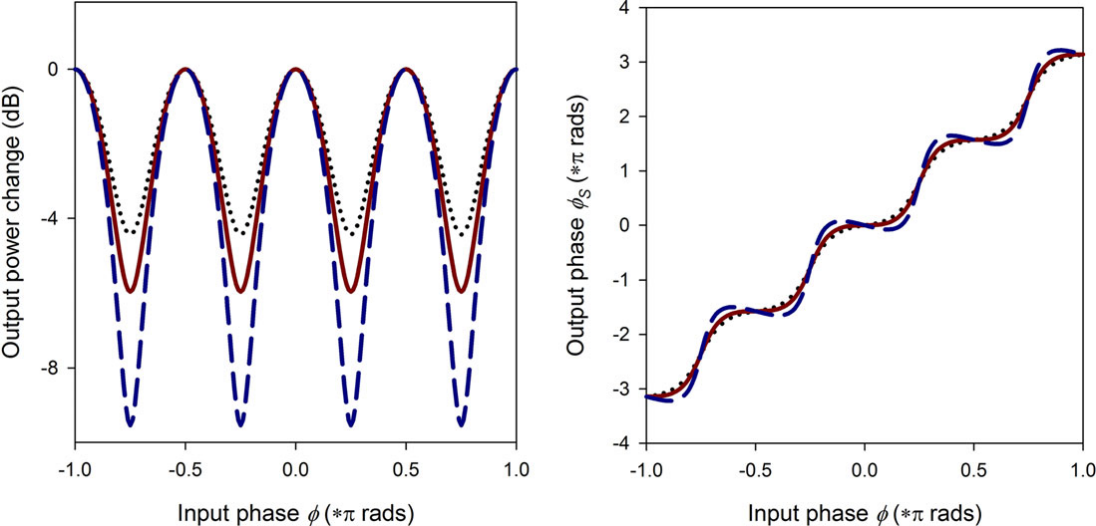
Evaluation of Eqs. (4) and (5) for *M* = 4, showing the transfer functions for various values of *m*. Dotted line is for *m* = 0.25, solid line *m* = 0.33, and dashed line *m* = 0.5.

Figure 10 shows the result of varying *m* in Eqs. (4) and (5) for *M* = 4 levels of phase modulation. The main outcome is that the phase transfer function exhibits a periodicity that matches *M*, but its steepness is a function of the weighing parameter *m* (and in fact, the optimum value of *m* is a function of *M*).

**Figure 11:**
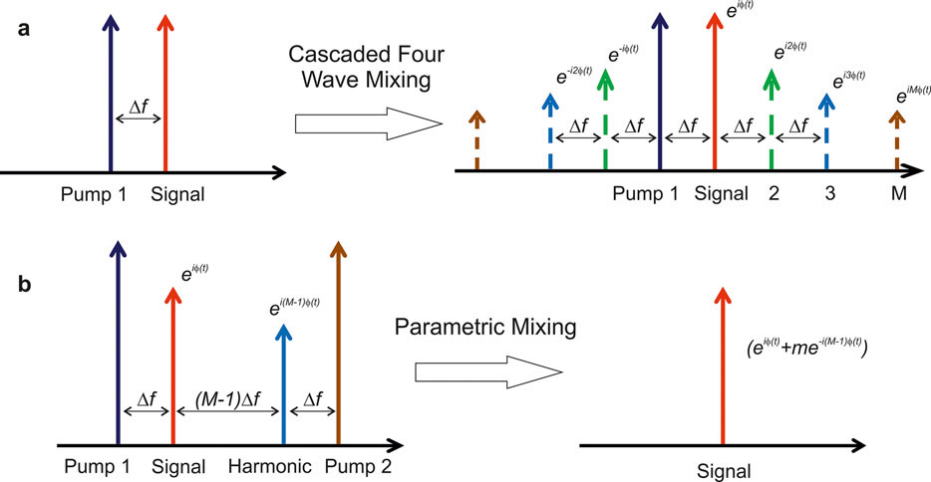
(a): Generation of higher-order phase-harmonics of a signal through mixing with a pump beam in a nonlinear medium; (b): Coherent combination of the signal with the (*M*-1)th harmonic using a dual-pump parametric process for the generation of a staircase phase response.

The physical implementation of this function would first require the (*M*-1)th phase harmonic to be generated from the signal using a cascaded FWM process with a frequency-detuned strong CW pump (Fig. 11a). By optimizing the phase matching and the strength of the nonlinear interaction, a spectral cascade of FWM products can be generated (Fig. 11a). Because of the phase relationship that exists between the waves involved in a FWM process, the comb of products possesses an overlying phase modulation that is a perfect integer multiple of the modulation present on the signal at the mixer input. Next, a second FWM process carried out using two pumps located symmetrically around the signal and phase harmonic (Fig. 11b) is required to coherently conjugate and add the (*M*-1)th phase harmonic to the signal.

**Figure 12:**
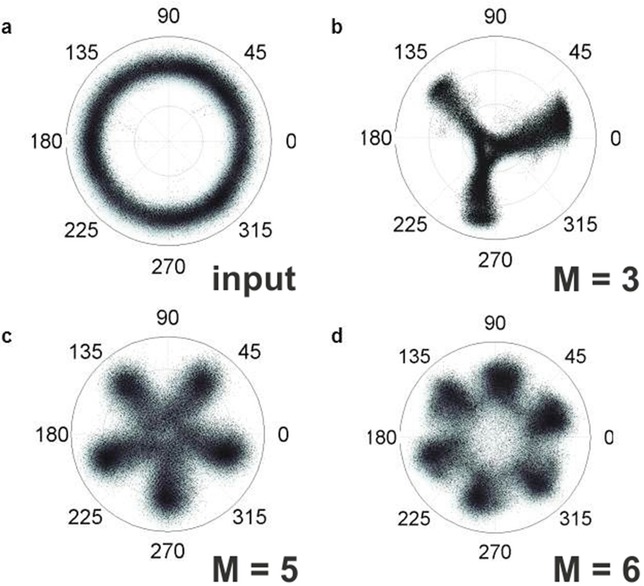
Experimentally measured signal constellation diagrams (a) before the quantizer, and after the quantizer for (b) *M* = 3, (c) *M* = 5 and (d) *M* = 6.

This process can readily be achieved experimentally, using a modification of the PSA set-up presented previously (see [32, 35, 36] for more details). Figure 12 shows the result of this process for three different values of *M*, namely *M* = 3, 5 and 6. To verify the operating principle, the signal phase was varied over 2π at a rate of 150 MHz in these experiments (see Fig. 12a). To switch from one value of *M* to another, the only requirement was to select the suitable phase harmonic, and optimize the pump and signal powers into the OPA. Figures 12b-d show successful phase quantization to a corresponding number of levels. As expected, the quantization was accompanied by a sinusoidal intensity response, whose depth decreases as *M* increases. This phase-to-amplitude conversion would be undesirable for some applications; this however can be eliminated in a number of different ways. Various possibilities include either the operation of the PSA in saturation as was described above in the case of BPSK signals (even though it is appreciated that the higher number of phase harmonics involved here would make this task challenging in many cases); subsequent regeneration of the signal amplitude in a high-dynamic range limiting optical amplifier, such as an injection-locked semiconductor laser [37]; or indeed, the involvement of a larger number of phase harmonics, which would alter Eq. (3), so that a more uniform amplitude response is obtained [38].

## 4. OPAs for broadband long-haul communication

It would be desirable to combine the OPA features of large gain bandwidth and low noise figure for insertion into long-haul communication systems suitable for handling the ever-increasing Internet traffic. To be competitive with current systems based on EDFAs and/or DRAs, such systems should be able to exhibit length × bandwidths products in excess of 100 Pb/s · km. Progress in that direction has been slow but steady. One reason for the lag is found in the issue of nonlinear crosstalk in the amplifiers, which is more severe than for EDFAs and DRAs. We first discuss this issue, and then describe the status of efforts in this area, both theoretical and experimental.

### 4.1. Nonlinear crosstalk

When a broadband optical spectrum, e.g. *N* wavelengths of a WDM system, is amplified in an OPA, the desirable Kerr nonlinear interaction with the pump(s) generates *N* idlers, which are an intrinsic part of the amplification process. We saw in Section 2 that in order to obtain a large gain bandwidth it is necessary to operate near the ZDW of the fiber, and to maintain low dispersion over a wide region. Unfortunately, the fact that dispersion is low also means that a number of undesirable nonlinear interactions will also be fairly well phase-matched, and may have a negative impact on the OSNRs of the signals. These interactions are: cross-phase modulation (XPM), cross-gain modulation (XGM), and high-order FWM.

XPM is also present in transmission fibers. It means that the time-dependent intensity of an amplitude-modulated channel induces modulation of the phase of all other channels. Since this effect depends weakly on dispersion, it may have a significant impact on phase-modulated signals.

XGM means that when a signal is amplified it receives photons from the pump, which is therefore intensity-modulated by this signal. This is also a very well phase-matched phenomenon, which must therefore be considered.

**Figure 13:**
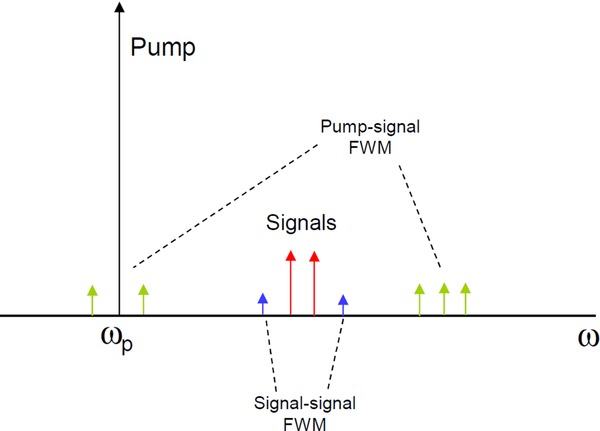
Partial output spectrum of a 1P-OPA, showing the location of undesirable signal-signal and pump-signal FWM terms.

The most complex detrimental effect corresponds to high-order FWM. This refers to FWM products originating from the three or four desired waves. Their study is complicated because the number of such terms is in principle infinite. This is because if a new wave is generated by two or three other waves, it too can in turn combine with other waves to generate new ones, etc. Fortunately, it is possible to classify such terms as being of first or second order, etc., and practically only the first few orders need to be considered. Even then, the situation can be fairly complex. This is illustrated by Fig. 13, which shows the approximate output spectrum of a one-pump OPA with just two signals at the input; the idlers are to the left of the pump, but are not shown for clarity.

Besides the signals, a number of FWM terms are shown: signal-signal FWM in blue, and pump-signal FWM in green.

Pump-signal FWM arises from mixing between the pump and one or two signals. Because the pump power is much larger than the signal power, the magnitude of these terms can be fairly large if they are well phase-matched, i.e. if the signals are close to the pump. A good thing about these terms is that they will not interfere with the signals if they occur in different spectral regions. This is the case in Fig. 13, where these terms fall either close to the pump, or on the far right side of the signals. However, if many more signals are added, so that they cover a significant bandwidth, then the bandwidths occupied by these FWM terms may begin to overlap with the signals. To avoid this situation, it is then necessary to limit the extent of the signal spectrum, which unfortunately means that the utilization of the potential OPA bandwidth will be reduced. In a 1P-OPA the use of a sub-octave signal spectrum will avoid such overlaps.

Signal-signal FWM arises from mixing between the signals themselves. For the two signals shown here there are only two such FWM terms, which do not overlap with the signals, and so are harmless. However, for three or more evenly-spaced signals, some resulting FWM terms do coincide with signals, and so introduce crosstalk. The number of such terms scales like *N*^3^, which becomes very large for typical WDM systems with tens of channels. Because of this strong *N*-dependence, and the inability to spectrally separate these terms from the signal spectrum, these terms present the most dangerous type of nonlinear crosstalk. This is an issue that must be addressed if fiber OPAs are to become competitive with the existing fiber amplifiers.

The recently introduced concept of hybrid parametric amplification may provide an additional tool for tackling the difficult problem of unwanted nonlinear effects in broadband systems [39, 40].

### 4.2. Phase-insensitive amplification

#### 4.2.1. Simulations

OPAs designed for optical communication systems have been the object of a number of theoretical and experimental investigations, and these have identified nonlinear crosstalk inside the OPAs as a potential performance-limiting factor. However, if we consider a typical long-haul communication system, we know that nonlinear effects taking place in the transmission links also introduce impairments. It is therefore important to investigate the relative impact of OPA and transmission fiber nonlinearities in a long-haul system using OPAs instead of EDFAs, to ascertain whether or not OPA nonlinear effects are more detrimental than the transmission effects.

**Figure 14:**
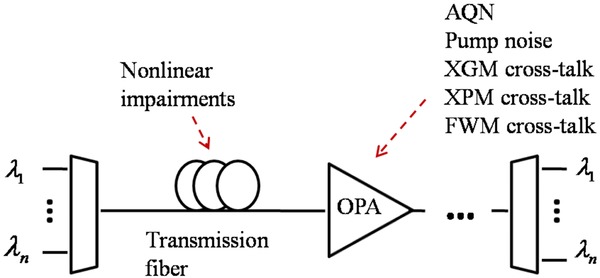
A WDM transmission link with inline phase-insensitive optical parametric amplifiers. AQN: amplified quantum noise, XGM: cross-gain modulation, XPM: cross-phase modulation, FWM: four wave mixing, OPA: optical parametric amplifier.

The first detailed investigation of this type was recently carried out. The potential performance of PIAs in long-haul WDM systems was investigated using analytical calculations as well as numerical simulations. Similar to other types of amplifiers, OPAs have several impairing effects on signals which should be taken into account to have a realistic performance evaluation. Figure 14 shows a WDM transmission link employing OPAs as inline amplifiers. Different degrading effects, which occur in such a transmission link, are shown in this figure.

**Figure 15:**
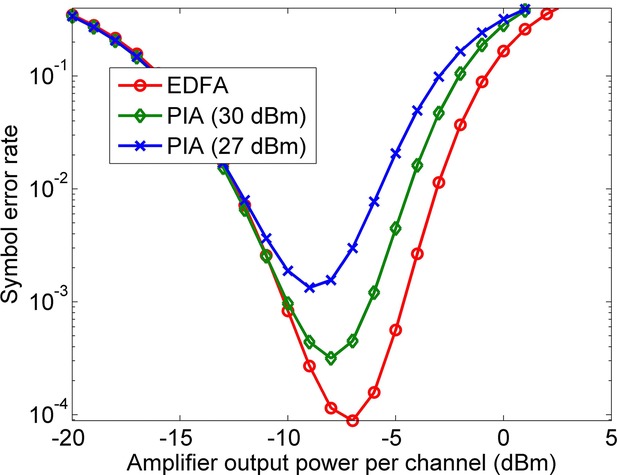
Symbol error probability versus the amplifier output power per channel for EDFA and PIA with different pump powers (100 × 28 GBd 16-QAM WDM Chs, 10 × 75 km fiber spans, 50-GHz grid).

The major noise sources in PIAs are the amplified quantum noise (AQN) and the pump transferred noise [41]. The pump noise is caused by RIN (relative intensity noise) of the pump laser and the ASE (amplified spontaneous emission) of the EDFA which is usually used to boost the pump power. The variance of AQN and the pump transferred noise can be analytically obtained [41, 42]. The major nonlinear degrading effects are the cross-gain modulation (XGM) and Kerr-induced effects such as XPM and FWM among channels, as described in the preceding section. Transmission fibers also cause nonlinear impairments such as XPM and FWM. These effects can be numerically modeled using a split-step Fourier method (SSFM) (p. 157 in [8]). However, SSFM simulation of a long-haul WDM system with many channels is time-consuming. Therefore, an analytical model was developed for this purpose [42]. The validity of the analytical model at high power levels was verified with SSFM simulations [42]. It was then used to evaluate the performance of a long-haul transmission system with the following specifications: 80 WDM channels on 50-GHz grid, 28 GBd 16-QAM per channel, 10 × 75-km fiber spans, 15-dB PIA gain, 4.5-dB noise figure amplifiers, standard single mode transmission fibers (SSMF). The inline amplifiers could be either EDFAs or PIAs. In Fig. 15 the resulting symbol error rate of this system is plotted versus the amplifier output power per WDM channel.

It is observed that EDFA and PIA lead to the same symbol error probability at low power levels, because of the same value of noise figure. Two PIAs with the same gain were investigated. One of them had a 27 dBm pump (500-m HNLF) and the other had a 30 dBm pump (256-m HNLF). The latter outperforms the former because of the lesser amount of FWM among channels in the shorter HNLF employed in the 30 dBm OPA [43]. It is also observed that the difference between the 30 dBm PIA and EDFA is low.

It was shown that the dominant cause of performance degradation at high power levels in WDM systems with small number of channels is the nonlinear effects of the transmission fibers rather than the nonlinear impairments due to the OPA [42]. However, if the number of WDM channels is large the nonlinear crosstalk due to the OPA becomes the dominant degrading effect. The reason is that the crosstalk in the HNLF occurs among all WDM channels due to the low value of dispersion in HNLF, while in the transmission fibers the crosstalk occurs mostly among neighboring channels due to the large dispersion value in the fiber. In order to transmit a larger number of WDM channels with low penalty, OPAs with large pump power levels should be used [43]. In Ref. [42] it is shown that using PIAs with 33 dBm of pump power, transmission of up to 170 × 28 GBd 16-QAM channels over 10 × 75 km fiber with a symbol error rate less than 10^−3^ is possible. The corresponding *B* × *L* product is 14,250 Tb/s · km, which is even comparable to the largest values reported in conventionally-amplified systems (16,500 Tb/s · km) [44]. Therefore, these simulations show that OPA-based long-haul systems may be realistic candidates for future high-capacity optical transmission links.

#### 4.2.2. Experiments

In this section we present the results of experimental demonstrations of the performance of OPAs at high data rates, as well as in a recirculating loop to test repeated amplification as required in long-haul systems.

##### A. 1 Tb/s WDM

This experiment was performed to amplify as many WDM channels as possible in a PIA, and to compare its performance to that of an EDFA [45].

The OPA was first evaluated in a back-to-back configuration. It was placed between the transmitter and receiver of a DWDM testbed and its performance was compared with a single-stage EDFA. The testbed contained 26 channels on the ITU grid with 100-GHz channel spacing starting from 1531.11 nm; the channels at 1537.40 nm and 1538.19 nm were missing. After multiplexing, the channels were modulated at 43.7 Gb/s by two LiNbO_3_ Mach-Zehnder modulators. The first modulator was driven by a 2^31^–1 pseudo-random binary sequence (PRBS) to generate a DPSK signal. The second was driven by a half-rate clock creating RZ-DPSK modulation with 67% duty cycle. The channels were decorrelated through 1.5-km of SSMF, and then launched into the EDFA or OPA.

The OPA used a single 35-dBm pump at 1572.5 nm. Its RIN at the HNLF output, measured with a 3 MHz resolution bandwidth, was −63 dB. Pump phase modulation by four RF tones was used for suppressing pump-induced SBS in the HNLF. The gain medium was a HNLF with *L* = 114 m, *γ* = 15 W^−1^km^−1^ and dispersion slope *D*_λ_ = 0.023 ps nm^−2^ km^−1^. The OPA was compared with an EDFA with a flat gain of 17 dB, NF = 5 dB and a maximum output power of 25 dBm.

The channel powers were leveled at the output of the amplifiers by adjusting the input channel powers. The total power at the OPA (EDFA) input was 1.5 dBm (−4.8 dBm). The signals were received using a tunable flat-top filter with a bandwidth of 0.6 nm to de-multiplex the selected channel, followed by a Mach-Zehnder delay interferometer and balanced photodiodes.

The performance of the OPA was evaluated by measuring BER versus power and OSNR at the receiver input. A 0.5-nm resolution bandwidth was used throughout for measuring OSNR, to ensure that the entire signal spectrum was included in the resolution bandwidth.

The normalized optical spectra of the transmitted channels at the output of the OPA and EDFA were measured. It was found that the noise level was different on either sides of the spectrum. This tilt in noise was the same for OPA and EDFA, and increased for increased total power or number of channels for the OPA. The tilt in the noise level for the EDFA is caused by the non-flat gain when it is used outside it optimum gain level. Also, by turning off channels in pairs across the band, e.g. 4 and 5, 16 and 17, different levels of noise were measured. Part of the OPA-induced noise is generated by nonlinear crosstalk due to FWM, which is greater at short wavelengths because of stronger phase-matching.

**Figure 16:**
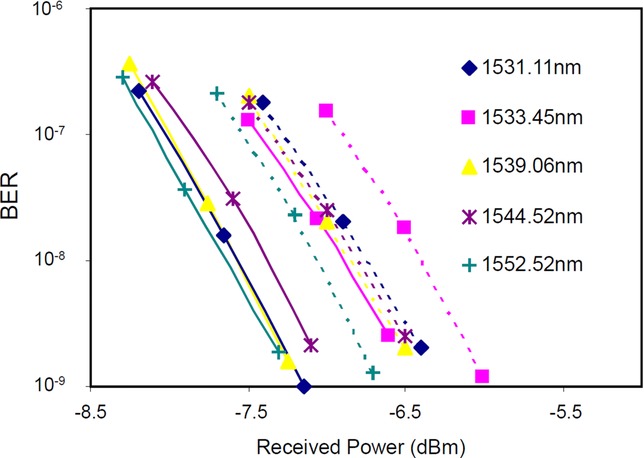
BER vs. received power of the data amplified by EDFA (solid line) and OPA (dotted line) at channels 1 (diamond), 4 (square), 9 (triangle), 12 (x), 16 (star), 20 (circle) and 26 (+).

With 34-dB input OSNR, the BER variations of channels 1, 4, 9, 12 and 26, versus the received power, are shown in Fig. 16. The accuracy on the received power level was 0.3 dB; therefore both amplifiers exhibited similar performance variation with channel wavelength. A 0.7-dB average penalty was found across the channels. The origin of the measured penalty at the OPA output is partly due to the coherent nature (FWM crosstalk) of the induced noise. Indeed, it was verified that the channels amplified by the OPA contained additional intrinsic noise, i.e. they had higher relative intensity noise (RIN). It originates from the pump and is independent of the signals characteristics.

These results show that OPAs can exhibit small power penalties compared to EDFAs when used for amplifying broadband WDM spectra with 1 Tb/s data rate, which is the highest rate achieved to date. The prospects are good for increasing this rate to multi-Tb/s by increasing the number of channels, using a higher-level modulation format, and possibly polarization-division multiplexing (PDM).

##### B. 0.64 Tb/s OTDM experiment

A category of signals where phase-insensitive fiber OPAs may be advantageous, is that of ultra-high speed optical time-division multiplexed (OTDM) signals. This technique enables the synthesis of optical signals with symbol rates beyond the current limitations of electronics by time-domain interleaving of short pulses modulated at base rates that are accessible to electronics. Symbol rates as high as 640 GBd [46], or even 1.28 TBd [47] have been demonstrated, enabling the generation of single wavelength channels with bit rates of 5.1 Tb/s [48] or even 10.2 Tb/s [49] using multi-level modulation and polarisation multiplexing. This requires the generation of pulses with full-width at half-maximum (FWHM) of the order of 300 fs, for which the transform-limited bandwidth is of the order of 12 nm. It is clear that the 35 nm bandwidth available from EDFAs would not enable the amplification of many wavelength channels, especially since a control pulse train at a distinct wavelength is also required at the receiver side in order to de-multiplex the signal down to the base rate (e.g. 10 GBd) by all-optical time gating techniques. Fiber OPAs may thus prove useful to such systems thanks to their potentially large bandwidths.

Compared to the parametric amplification of WDM signals, an OTDM signal would not suffer from inter-channel crosstalk, which in turn could relieve the pump power versus HNLF length trade-off. Furthermore, when operated in saturation, fiber OPAs enable amplitude equalization [50], which may provide the additional benefit of regenerative amplification.

**Figure 17:**
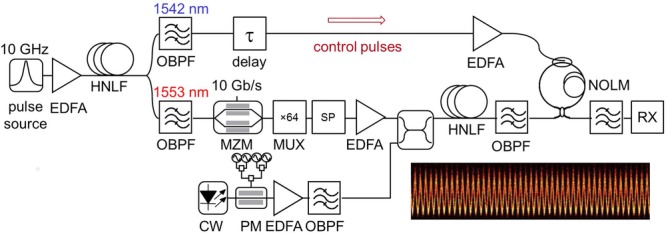
Experimental set-up for parametric amplification of a 640 Gb/s OTDM RZ-DPSK signal.

The parametric amplification of a 640 Gb/s signal obtained by time-multiplexing of 620-fs short optical pulses modulated in the DPSK format at 10 Gb/s has been recently demonstrated [25]. The experimental set-up is represented in Fig. 17.

Two 10 GHz short-pulse trains centered at 1542 nm and 1553 nm are obtained by filtering a supercontinuum seeded by a 10 GHz optical pulse source in a dispersion-flattened HNLF. The pulse train at 1553 nm is modulated at 10 Gb/s in the DPSK format with a 2^7^-1 PRBS in a Mach-Zehnder modulator (MZM) before being multiplexed to 640 Gb/s in a passive fiber delay line multiplexer. The high-speed signal is then injected into a single-pump fiber OPA made from a 500-m long HNLF with ZDW = 1569 nm, *D*_λ_ = 0.016 ps nm^−2^ km^−1^, *γ* = 11.4 W^−1^km^−1^, and *α* = 0.7 dB km^−1^. Phase modulation with three frequency tones is imposed on the pump to increase the SBS threshold. After amplification and band-pass filtering, the signal is de-multiplexed to 10 Gb/s by exploiting cross-phase modulation from the 10 GHz control pulse train at 1542 nm in a nonlinear loop mirror (NOLM) made from a 50-m long HNLF. Implementation details can be found in Ref. [25]. The de-multiplexed signal is finally detected in a pre-amplified receiver with a 1-bit delay fiber interferometer followed by balanced detection.

**Figure 18:**
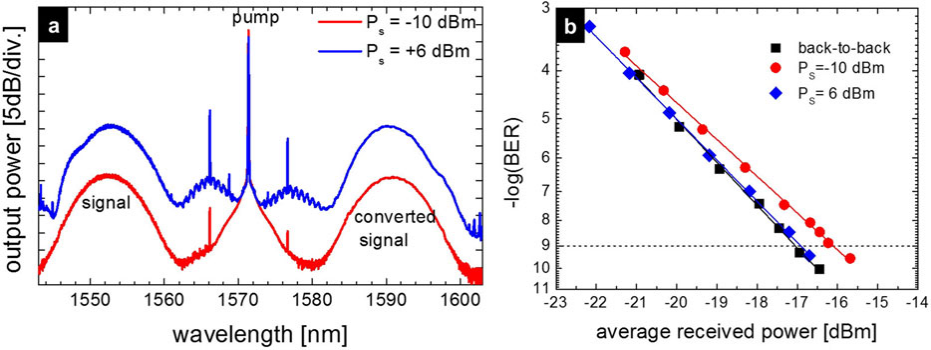
(a) Spectrum at the HNLF output for the amplification of a 640 Gb/s OTDM signal with pump power of 27 dBm and signal input power of -10 dBm and 6 dBm. (b) Corresponding BER curves for one of the 64 OTDM channels.

One key issue for the amplification of such short pulse signals is to engineer the OPA gain profile so that it does not induce significant spectral shaping. In a single-pump fiber OPA the available degrees of freedom are the choice of the pump wavelength as well as the exploitation of saturation, which can be used to flatten the gain bandwidth. In the conditions of the experiment, a flat gain over a bandwidth sufficient to accommodate the 6 nm FWHM spectral width of the signal could be obtained for a pump wavelength of 1571.5 nm and a pump power of 27 dBm at the HNLF input. For such broadband signals, one should pay attention to avoid overlap between the signal spectrum and the first high-order idler generated on the same side of the pump as the signal. Fig. 18(a) represents the spectrum at the output of the HNLF for signal input power values of −10 dBm and 6 dBm, corresponding to net gains of 15 dB (unsaturated) and 13.3 dB, respectively. The corresponding BER curves are represented in Fig. 18(b) for one of the 64 multiplexed channels. A penalty of 1 dB compared to back-to-back is measured for −10 dBm input power, which can be reduced to about 0.1 dB when exploiting saturation.

The high quality of these results indicates that fiber OPAs have the potential for amplification of high-speed TDM signals in optical communication links.

##### C. Recirculating loop: Transmission over a fiber OPA-amplified link

In spite of the potential advantages of phase-insensitive fiber OPAs, very few studies on their use as in-line amplifiers have been reported. A re-circulating loop experiment involving fiber OPAs was presented in Refs. [45, 51]. However, the OPA was placed outside the loop and its operation as in-line amplifier was emulated by degrading its input signal-to-noise ratio. The use of fiber OPAs as in-line amplifiers was also evaluated using an analytical additive noise model [42], as detailed in Section 4.2.A.

**Figure 19:**
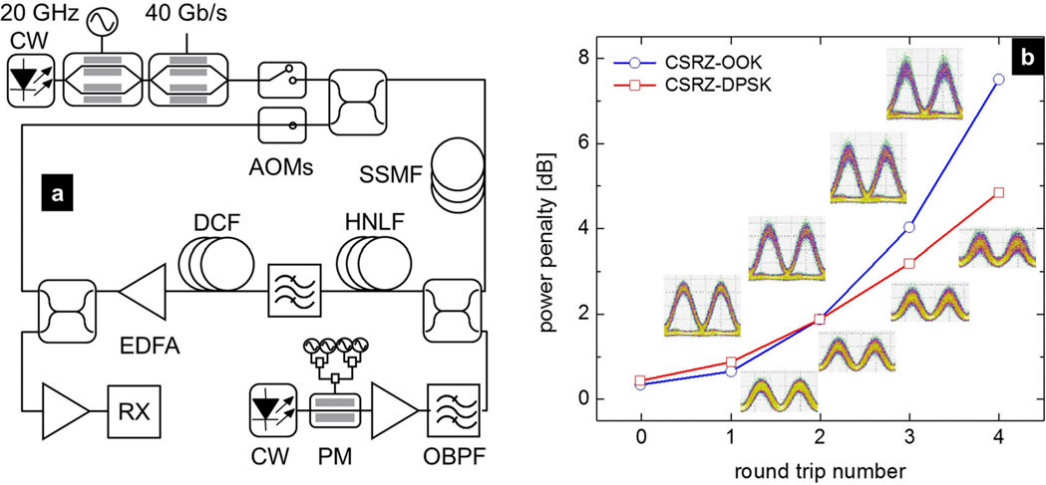
(a) Re-circulating loop set-up for cascaded single-pump parametric amplification. (b) Measured power penalty (BER = 10^−9^) as a function of number of round trips for the CSRZ-OOK and CSRZ-DPSK formats at 40 Gb/s. The corresponding eye diagrams are represented as insets.

Some preliminary experimental results describing the use of a single-pump fiber OPA as an in-line amplifier within a dispersion-managed span in a re-circulating loop have been presented in Ref. [52]. The re-circulating loop consisted of a single-span of 80-km SMF, followed by a single-pump fiber OPA, and a matching length of 13-km dispersion compensating fiber (DCF), as illustrated in Fig. 19(a).

An EDFA was used to compensate the excess loss due to the coupling to and from the loop, including the loop switch built from acousto-optic modulators. The OPA used a 500-m long HNLF with ZDW = 1550.4 nm, *D_λ_* = 0.0185 ps nm^−2^ km^−1^, *γ* = 10.7  W^−1^km^−1^, and *α* = 0.7 dB km^−1^, resulting in 20 dB on-off gain for a pump power at the HNLF input of 28 dBm. The transmission performance of the fiber OPA-amplified link was evaluated as a function of the number of round trips for both carrier-suppressed return-to-zero (CSRZ) on-off keying (OOK) and DPSK formats at 40 Gb/s. The resulting power penalty at a BER of 10^−9^ is shown as a function of the number of re-circulations in Fig. 19(b), together with corresponding eye diagrams. The good preservation of the pulse shape suggests the system was limited by noise accumulation. The well-known increased robustness of the CSRZ-DPSK format is also visible. However, these results are practically limited by the fact that no polarization-independent fiber OPA scheme was implemented (such as e.g. in [53, 54]) and the loop was operated up to a limited number of round trips by identifying a stable principal state of polarization. Unfavourable noise accumulation due to the fiber OPA was also responsible for the relatively high measured penalties.

This experiment represents the first experimental demonstration of periodic amplification by a fiber OPA in an optical transmission link. The 372-km distance reached exceeds the scale of typical metropolitan networks, and it bodes well for extending such experiments on the scale of long-haul networks.

### 4.3. Phase-sensitive amplification

#### 4.3.1. Introduction

Perhaps the most intriguing property of PSAs is the possibility to approach a 0 dB NF for the in-phase component [55], unlike conventional amplifiers which have a quantum-limited NF = 3 dB. The first fiber based PSA was demonstrated in 1990 [56] and the first sub-3dB NF in 1999 [57, 58]. Later WDM compatibility of PSAs was also demonstrated [59, 60]. Accurate NF measurement techniques were presented and resulted in detailed understanding of the noise properties of fiber PSAs [61, 62]. In this review, we focus on the use of HNLFs as gain medium in phase-sensitive fiber-optic parametric amplifiers (PS-FOPA) implemented in a single-pump non-degenerate idler configuration, thus having three input waves; pump, signal and idler. In some sense, a PSA has similarities to a coherent receiver, in which an LO wave and signal wave add coherently. However, in a PSA, it is the signal and idler waves that add coherently. One could thus view it as a “coherent amplifier”. As such, the in-phase components of the waves add constructively, while out-of-phase components beat destructively. For deterministic, correlated signals on these waves, these amplifiers can be tuned to provide constructive addition. However, for uncorrelated, stochastic components, such as independent noise sources, the addition is incoherent. Effectively half the noise will add constructively while the other half cancels destructively. This difference between the beating of correlated signal and un-correlated noise components gives rise to the low-noise amplification achievable in PSAs. The lowest NF reported to date at high gain (1.1 dB at 26 dB gain, i.e. well below the classic 3-dB quantum limit) of any high-gain optical amplifier was demonstrated in Ref. [63].

Here we review some basic properties of PSAs and, in particular review recent progress toward practical use in real transmission systems. While challenges remain, recent results show clear performance improvement when comparing to systems utilizing EDFAs as amplifiers.

#### 4.3.2. PSA concepts

Key building blocks of any fiber OPAs are: (i) the specifically-designed HLNF that should not only have low dispersion near the operating wavelength, but also low attenuation and be easy to splice to other types of fibers; (ii) the high-power single-frequency pump laser with low intensity noise. In addition, for PS-FOPAs idler(s) satisfying appropriate phase conditions have to be generated and, in practice, phase-locked loops (PLL) are often needed to maintain optimal conditions in the presence of environmental disturbances.

In the single-pump, non-degenerate idler configuration, which has been the focus of this work, the signal and idler wavelengths are symmetrically located around the pump. Multiple signal-idler pairs can interact with the pump making the scheme compatible with the use of several DWDM channels. Idler waves must not only have the correct optical phase but also contain the encoded data from the corresponding signal. If an idler contains a phase-conjugated copy of the signal data, the PSA will operate with any modulation format, while if not, it will only amplify one quadrature [64]. This is suitable for e.g. binary PSK signals, which would then not only be amplified but also phase-regenerated. In our case, we simply use a PI fiber FOPA to generate a stable idler (which we call ‘copier’, see e.g. Fig. 21). The control of the relative phase relation among the different waves can be implemented with a feedback control loop (by e.g. maximizing the PS gain). The phase-sensitive amplification is dictated by the relative phase among the interacting waves. The input/output optical field relation of a PS-FOPA at perfect phase matching is given by [65]:

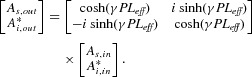
(6)

**Figure 20:**
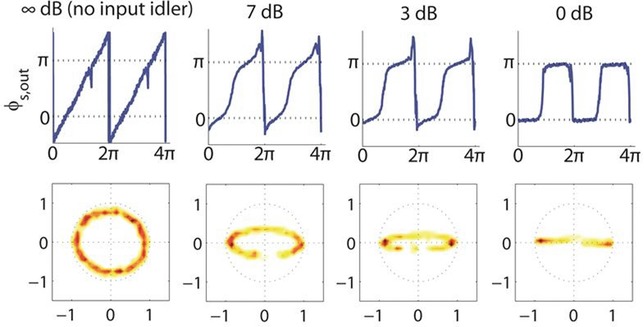
Signal phase (top) at PSA output versus input phase for different idler powers and corresponding complex plane constellation diagrams (bottom), in which the x- and y-scale represent normalized real and imaginary parts of the electric field.

**Figure 21:**
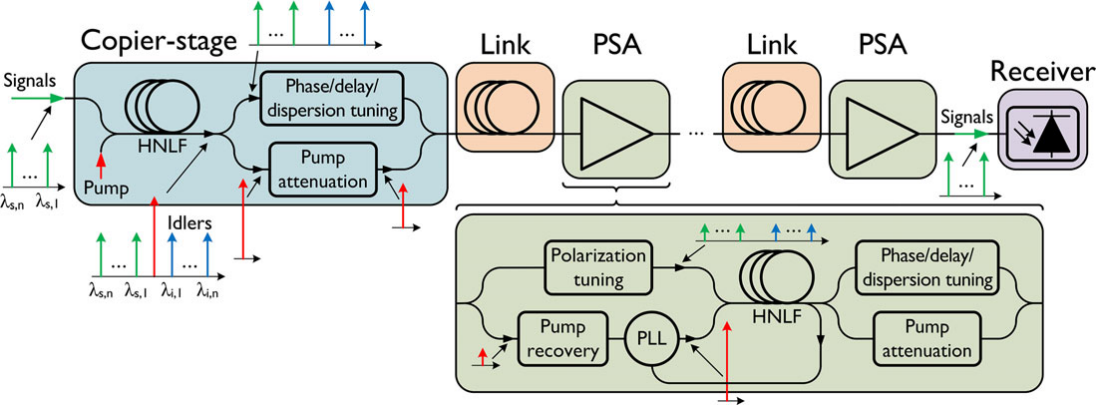
Illustration of the use of PSAs in DWDM transmission links.

Here *L*_eff_ is the effective length of the fiber, given by

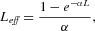
(7)
where *α* is the fiber power attenuation coefficient. 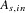 and 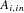 (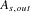 and 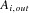) are the signal and idler phasors at the input (output). Clearly, in the case of no idler present at the input, one will be generated, and the signal phase is conserved.

In the case of PS amplification, we assume that the signal and idler input powers are equal, and so we have 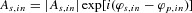 and 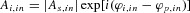. Solving Eq. (6) one finds the expression for signal gain as

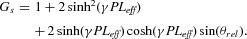
(8)

The quantity *θ_rel_* is the relative phase among the three waves. In the case discussed here, it is given by 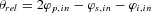 with subscripts *p*,*s*,*i*, representing the absolute phase of the pump, signal and idler, respectively. It is seen from this equation that only signal phases satisfying *θ_rel_* = π/2 are fully amplified while those resulting in *θ_rel_* = −π/2 will be parametrically attenuated.

For simplicity, we set 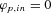 as a frame of reference and find the signal output phase dependence on the input phase as

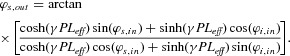
(9)

Note that in the no-gain limit Eq. (9) reduces to 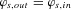, while for high gain, the output signal phase can only assume 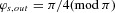.

This is illustrated in Fig. 20, showing experimental results of output-input phase relations and constellation diagrams when varying the ratio of idler to signal power [66].

As seen from Eq. (6), if the input idler is the conjugate of the signal (e.g. generated in a FWM-based copier), both terms in the output signal field are directly proportional to the input signal field. The dependence on the absolute signal phase will then be canceled in the relative phase, θ_rel_. This is the explanation of the modulation format independence of the copier-PSA system used below for transmission studies.

#### 4.3.3. PSAs as in-line amplifiers in transmission links

Clearly, if 0 dB NF amplifiers could be used as in-line amplifiers, it would be beneficial as the overall transmission link NF would improve. In fact, it has been shown that the link NF can be improved by up to 6 dB compared with using ideal EDFA amplification, if implemented as a cascade of a copier stage (serving to generate idlers) and assuming that the noise from the copier can be neglected in the output signal (which is the case for long-haul transmission) [67]. This comes, however, at the expense of propagating the idlers occupying useful spectrum. The link NF is thus ideally entirely limited by the span losses. For any given modulation format, this concept allows the signal power in the link to be reduced by a factor of four compared with the traditional approach, thus significantly reducing the degrading impact of transmission fiber nonlinearities.

Figure 21 illustrates the concept that we have considered [68]. The copier was straightforwardly implemented by using the FWM process in a HNLF and requires no active control. It should be understood that the only purpose of the idlers is to facilitate noiseless amplification of the signals in the in-line PSAs (as well as for mitigation of impairments caused by transmission fiber nonlinearities as will be discussed below), and once the signals and idlers have reached their destination, only the signal waves are recovered with traditional direct detection or coherent detection receivers. The figure also illustrates some details in terms of the in-line PSA implementation. For practical purposes, the pump wave needs to be attenuated prior to transmission through the link in order to avoid SBS in the transmission fiber. With injection-locking, penalty-free pump recovery was demonstrated down to −30 dBm of pump power into the recovery system which provides plenty of margin for spans over 100 km [69]. A PLL is also needed to ensure proper phase relation. The signal and idler waves can be separated from the pump wave and combined with the amplified wave from the slave laser with low-loss WDM couplers ensuring that the “black-box” NF of the PSA is kept very small. There is also a need for periodic dispersion, time delay, polarization, and static phase control among the waves and this can be implemented at the span input, thus not impacting the link NF.

**Figure 22:**
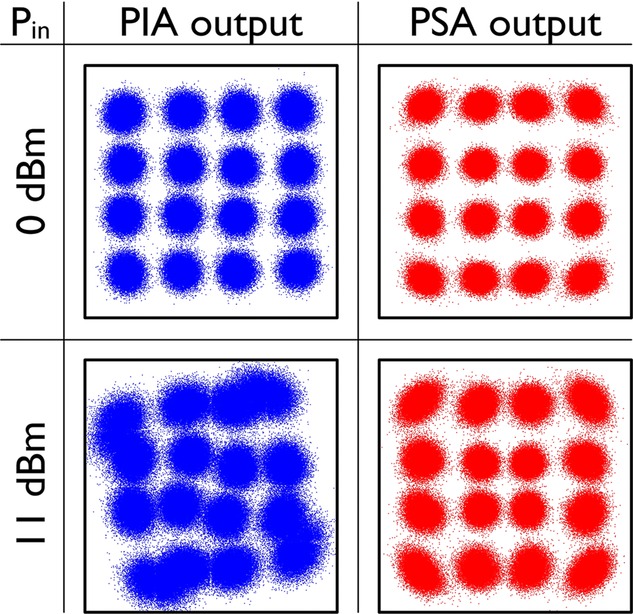
Constellation diagrams of 40Gbit/s 16-QAM signal after 105 km transmission at low power (0 dBm – top row) and high power (11 dBm – bottom row) for phase-insensitive (left) and phase-sensitive (right) amplification.

In terms of benefits with this approach, the 6 dB link NF improvement, which was confirmed experimentally in Ref. [67], can be used to increase overall reach by a factor of four or increase the span length, or increase the number of bits/symbol in multilevel modulation formats by approximately 2 bits/symbol per polarisation (as the OSNR needed increases approximately by 3 dB for each added bits/symbol [70] or a combination of the above. Recently, we conducted a direct comparison of a PS-FOPA versus an EDFA as preamplifier in a real fiber transmission experiment over 80 km with 10 GBd QPSK signals [71]. The results showed 2.6 dB better sensitivity (at BER = 10^−4^) for the PSA when comparing with the EDFA in which two optical WDM channels carrying identical data were detected simultaneously (to emulate the PSA case of signal plus idler carrying the same data). Our experiments further show a large degree of tolerance against nonlinear impairment caused by SPM in the transmission fiber [72]; a 3dB Q-penalty reduction relative to the case when not using the signal/idler superposition i.e. the phase insensitive mode was measured. This is a result of the coherent superposition of the signal/idler waves in which nonlinear phase rotation is partially cancelled. The dispersion map plays a key role in its effectiveness, however. Recent results, using 40 Gb/s 16-QAM formats shown below in Fig. 22 illustrate the mitigation of nonlinear phase rotation [73]. The PSA provides much less distortion, in addition to providing lower noise, thus extending the system reach. Similar nonlinear mitigation has also recently been observed by co-propagating a signal and conjugated idler in a link and performing superposition after detection in a receiver by using digital signal processing [74, 75]. The PSA approach, however, performs this all-optically.

Recently, we also demonstrated a record sensitivity (55 photons/bit at BER = 10^−9^) for on-off keying modulation at 10 Gb/s by using a PS preamplifier [76], breaking a 17-year old record using an EDFA-based preamplified receiver [77].

#### 4.3.4. Discussion

While only half of the available low-loss transmission band in the transmission fiber is available for signal transmission due to the need for idler transmission, PSAs allow for an interesting trade-off between bandwidth usage and noise/nonlinearity mitigation performance. In addition, fiber OPAs have been demonstrated to have an effective gain bandwidth larger than 100 nm [78], thus by far surpassing the available bandwidth in EDFAs. Consequences of the fact that periodic in-line dispersion compensation is needed should also be analysed. There are several challenges to overcome before the full prospects of PS-FOPAs can be understood and implemented in real systems, not necessarily for telecom applications. In many experimental demonstrations, active mitigation of SBS by use of pump phase modulation has been used. This degrades the performance of fiber OPAs in several ways and should ideally be avoided. We have avoided this problem by using stretched fibers and in-line isolators [79].

### 4.4. Mid-span spectral inversion

**Figure 23:**
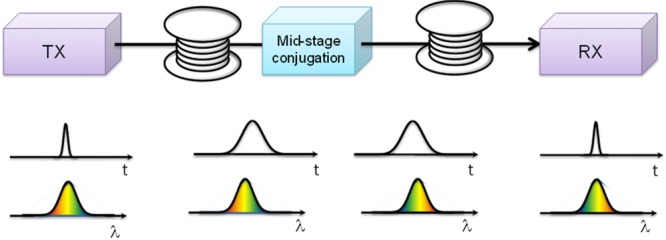
Mid-span conjugation principle: channel spectrum is spectrally inverted (conjugated) in the middle of the link and, in the case when the signal bandwidth is small, results in near-ideal reconstruction of the launched waveform.

Recognized earlier [80] for its inherent ability to compensate not only dispersion-induced but also nonlinear penalties, mid-span optical conjugation was a subject of multiple demonstrations in the past [80, 81]. While a conjugation node can be easily constructed in the case when signal bandwidth is small and only a single channel is transmitted, current and near-future high-capacity transmission will not satisfy either of these requirements. Indeed, in addition to the WDM nature of any future lightwave link, the physical bandwidth associated with a single channel is expected to be sufficiently large to merit consideration of higher-order dispersion penalties in the link. Figure 23 illustrates the basic principle behind mid-span spectral inversion in which a single, quasi-monochromatic channel undergoes spectral inversion to reconstruct a near-ideal transmitted waveform.

**Figure 24:**
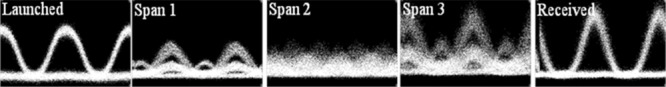
WDM transmission compensated by polarization-invariant mid-link compensator: typical channel waveform (10Gb/s) at launch, and interim spans of the link [81].

Even when the bandwidth of the channel is sufficiently small to neglect distortion from higher-order dispersion, practical transmission must include more than a single channel. In this case, the phase conjugation node must also be polarization invariant to accommodate random polarization states corresponding to distinct channels entering the module. While a single-pump parametric mixer can, at least in principle, be constructed to provide the desired level of polarization invariance, such approach requires signal splitting and complicates signal compensation. In contrast, a two-pump parametric mixer, realized in low-birefringence HNLF can be used to design a polarization-insensitive conjugator without any polarization or combining elements. Figure 24 illustrates MSSI dispersion-compensation results obtained with a WDM conjugated link that relies on a two-pump, orthogonally-polarized parametric mixer.

While relatively simple, this approach will not work in cases when the signal bandwidth becomes large. To understand the limits of mid-span conjugation, consider the transmission over a fiber possessing a positive *β_3_* (such as SSMF). In the case when a transform-limited pulse is launched, it will acquire dispersion-defined chirp and enter the mid-span conjugator. An ideal, two-pump conjugator will spectrally invert the pulse spectral envelope and phase, perfectly compensating all even-order dispersion terms, but allowing odd-order dispersion to accumulate further in the second half of the link: at the end of the span, the accumulated phase rotation is 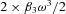. As a consequence, when pulse duration is sufficiently short, third-order dispersion will induce non-negligible penalty that scales with the channel rate. In the case of a 640 Gb/s TDM channel, the characteristic length corresponding to third-order dispersion penalty in SSMF is only 1.5 km, thus eliminating such conjugation scheme from practical consideration. To address this limitation, it is necessary to compensate odd-order dispersive terms outside the conjugator node, by adding a fiber segment that has an opposite sign for the higher-order (odd) dispersive term. The first half of the link needs to be designed specifically to cancel odd-order dispersion by combining SSMF and DCF types and guarantee that the phase rotation in SSMF, 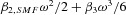, is being matched by the phase evolution in DCF, 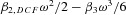. Mid-span conjugation will invert the quadratic rotation and result in cancellation at the end of the link; the odd-orders between SSMF and DCF will cancel even in case when conjugation is not performed.

**Figure 25:**
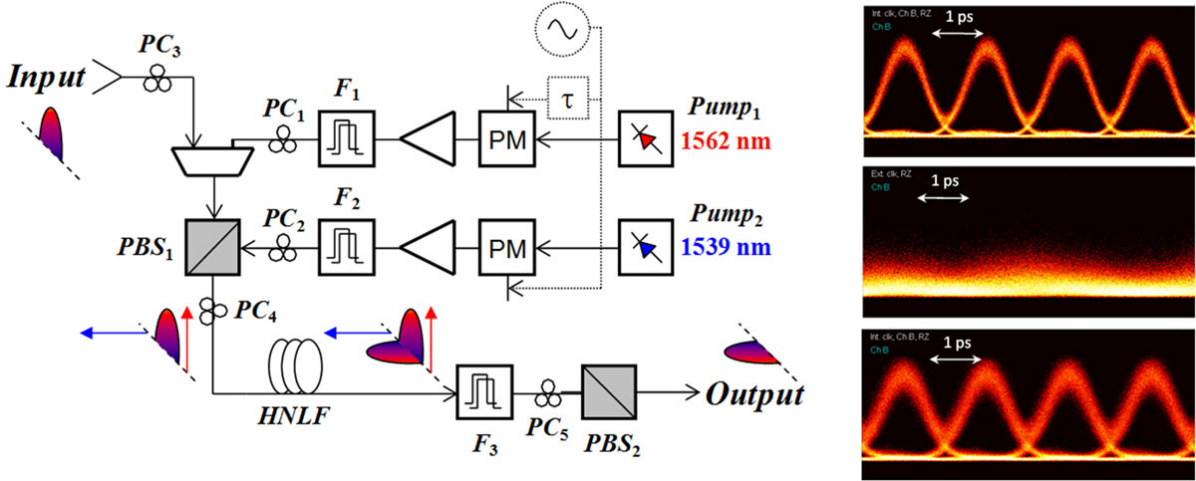
(Left) Polarization-invariant, frequency-degenerate conjugator constructed with orthogonally multiplexed pumps; (Right) 640 Gb/s RZ-OOK channel compensated by the conjugation node and received after 100-km SSMF link: top- launched; middle- mid-span; bottom- received [82].

This approach was recently demonstrated by transmission of a 640 Gb/s signal possessing optical bandwidth of 15 nm [82]. The channel was sent over a 100-km SSMF link, and a two-pump polarization-invariant conjugator was placed mid-span. The use of a two-pump conjugator architecture has an additional advantage as it also results in a frequency-degenerate idler (i.e. the idler has the same frequency as the signal) that eliminates any need for additional compensating steps that would be necessary due to finite fiber slope. The architecture of the conjugator, shown in Fig. 25, relied on orthogonally-polarized pumps separated by 31 nm, and allowed direct polarization stripping of the power-equalized idler wave. Remarkably, the entire compensation scheme resulted in a low-error (BER < 10^−9^) 640 Gb/s link without any need for electronic correction (FEC).

## 5. Challenges

While fiber OPAs present a number of features of interest for optical communication, optimizing these features simultaneously in a single device has proved difficult, for a variety of reasons. In Section 4.1 we discussed the subject of nonlinear crosstalk in broadband networks. Here we discuss some of the main challenges for fiber OPAs, and what developments are desirable for addressing them.

### 5.1. Stimulated Brillouin scattering

SBS is a nonlinear acousto-optical effect, which is present in all materials. It is mediated by the excitation of acoustic waves. The backscattered light is downshifted by about 10 GHz from the pump. SBS has a high gain coefficient, approximately 100 times larger than the Kerr coefficient. Hence if it is not modified in some way, it will reflect essentially all the pump power when it is increased above a certain value. Therefore, some mechanism must be introduced to reduce the effectiveness of SBS by a large amount (i.e. by 10–20 dB). Several techniques are known to suppress SBS, and some may be combined for improved suppression.

An important technique is the use of phase modulation (PM) of the pump to broaden its spectrum. This technique is very convenient because it works with any fiber, and does not require any permanent modification of the fiber. However, pump PM leads to a modulation of the phase-matching conditions because the pump frequency is also modulated, and this in turn modulates the gain. This is highly undesirable in communication systems as it introduces noise. In addition, pump PM is generally not compatible with maintaining the very precise phase relationships necessary for PSA operation. For these reasons, it would be highly desirable to avoid this approach for high-performance optical communication.

Another method consists in applying a longitudinal stress distribution along the fiber. This has the effect of broadening the overall SBS gain spectrum, and of reducing the peak gain. However since this occurs in a static manner, the preceding problems are entirely avoided. Unfortunately this method has its own disadvantages, namely: stress also modifies dispersion, and this affects the shape of the gain spectrum; the stress distribution must be applied permanently to the fiber, preventing its reuse under different conditions; finally the SBS gain reduction achievable in this manner is about 10 dB, significantly smaller than by pump PM. In spite of these limitations, this type of fiber is now commonly used in PSA experiments.

Inserting one or more isolators in the fiber of an OPA blocks the light reflected by SBS and therefore the SBS threshold for the whole fiber is increased to the threshold value for the longest segment isolated in this manner. Hence if *N* equally-spaced isolators are inserted, SBST is increased by 10log(*N*) in dBs [83]. The main difficulty with this approach is that each isolator has a finite insertion loss, and the accumulation of these losses generally limits their useful number to just a few. The largest number reported to date is 3 [76]. In this particular work, isolators were used in conjunction with stress, yielding a large increase in SBST, sufficient for obtaining a high PSA gain (21 dB) without resorting to PM. Further developments along these lines would help avoiding PM, thereby avoiding various penalties that accompany pump PM, and facilitating the use of phase-modulated signals in particular.

### 5.2. Gain spectra

To date the broadest CW OPA gain spectra have been obtained in silica-based HNLFs. This was accomplished in part thanks to improved designs of the index profile by manufacturers, which led to the fabrication of fibers with low values of *β*^(4)^, essential for obtaining broad gain spectra (see Section 2). However, while these spectra can be several hundred nanometers wide, they are not very flat. Hence in order to be useable in wideband long-haul systems, gain flattening filters would need to be introduced at suitable locations. Of course this is not a new problem in optical communication, because the gain spectra of EDFAs also need to be flattened periodically. This is generally accomplished by using two-stage EDFAs, with a filter placed in the middle. In principle the same strategy could be used for OPAs, however special care will need to be exercised to avoid spectral modifications introduced by the filter dispersion, including that of the connecting fibers.

A difficulty with OPAs, not present in EDFAs, is that the shape of the gain spectrum may vary significantly from one fiber to another, even though they are nominally identical, i.e. come from the same spool. The reason for this is the lack of longitudinal uniformity, which leads to random spatial modulation of the dispersion parameters. With such spectral variability, it would be necessary to tailor and implement each filter individually, a costly proposition. A novel fiber design greatly reducing the sensitivity of fiber dispersion to diameter fluctuations was recently introduced [84]. Such fibers stabilize the shape of the gain spectrum, however to date only narrowband spectra have been obtained.

For broad OPA gain spectra Raman gain can also cause significant spectral distortions. In principle these can be corrected with filters as in the preceding. However the Raman gain also causes an increase in the OPA NF, particularly in the region of maximum gain. This effect will need to be carefully taken into account in the design of broadband transmission systems.

### 5.3. Polarization independence

In their simplest forms, OPAs have a gain which depends on the relative states of polarization (SOPs) of the input waves. In particular, if the SOPs of the input signals are different, or if they vary in time due to thermal fluctuations of a transmission line, the gain is not always equal to its optimal value. This is an impairment which will degrade system performance. This issue is particularly important as it is highly desirable to use polarization-division multiplexing (PDM) in order to increase the spectral efficiency of the transmission systems. Some techniques have been introduced for dealing with polarization independence.

For 1P-OPAs the use of polarization diversity has been demonstrated. In this approach the signal input SOP is decomposed into its *x* and *y* components by means of a polarization beamsplitter (PBS). These are then separately amplified by two identical OPAs, and recombined by means of another PBS. The output power is then independent of the input SOP. This technique can be conveniently implemented by using a single fiber loop, and amplifying the two SOPs separately as they are made to travel in opposite directions. While this approach has the merit of being relatively economical, it has the disadvantage that some nonlinear crosstalk can occur at high gain between the two directions, due to XPM or SBS. To avoid this one would need to use two separate OPAs, which has its own disadvantages.

For 2P-OPAs, it is also possible to use the same polarization diversity approach as above. But another possibility arises due to the presence of two pumps. If their SOPs are orthogonal, then the signal gain is polarization-independent [54]. This approach also has the advantage that it can result in a gain spectrum which is flatter than for a 1P-OPA. A drawback is that the total pump power required for a given gain is about three times larger than if the pump SOPs are parallel; this in turn makes SBS suppression more difficult. Nevertheless, in spite of these difficulties this technique offers a fairly straightforward way of achieving the polarization independence required for practical systems. (See Section 4.4 for an example of implementation.)

While silica-based HNLFs are currently the best platform for fabricating high-performance OPAs, future developments in the area of highly-nonlinear non-silica fibers could eventually lead to improved OPA systems. Fibers with *γ*'s 1000 times larger than HNLFs have been demonstrated, however to date their relatively high losses and low damage thresholds have prevented them from being useful for CW fiber OPAs. Improvements of these parameters could eventually lead to more compact OPAs with low pump powers, and possibly to the development of pump resonators and their associated benefits [85].

Finally, the development of high-power high-OSNR pumps would be highly desirable. Currently high-power pumps are obtained by amplifying a high-OSNR seed by EDFAs. Such amplification degrades the optical OSNR, because it is very difficult to filter out the ASE generated by the EDFAs in a very narrow band about the high-power pump. This residual ASE in turns leads to signal degradation via FWM. To improve this situation, one would need to do one of the following: (i) develop narrowband filters that can operate at high power, and with low loss; (ii) develop narrowband lasers which directly generate a high output power, without requiring further amplification. While some of the issues presented above are more pressing, this problem of pump quality should eventually be addressed as well.

The challenges presented in this section are in principle probably amenable to individual engineering solutions. Of course an additional challenge will remain, which is that of finding solutions that are mutually compatible, so that they can be integrated into practical system designs suitable for testing in communication systems.

## 6. Conclusion

A major driver for the development of fiber OPAs has always been the possibility of eventually utilizing their unique properties to improve the performance of fiber optic communication systems. After early work in the 1980s, a good theoretical understanding of their capabilities and limitations was developed in the 1990s. The late 1990s and early 2000s saw a number of experiments performed to verify their capabilities, with respect to basic features such as gain bandwidth, polarization dependence, noise figure, etc., as all these aspects are essential for the design of communication systems. From the mid-2000s until now, the focus has turned increasingly toward sub-system and system studies, incorporating a few of these basic features to attack engineering problems directly relevant to communication systems. A major example of this is the European research program PHASORS, which concentrated on the use of PSAs for signal regeneration, as well as for low-noise amplification. In parallel, the issue of nonlinear crosstalk was tackled to permit the amplification of a large number of channels through a single OPA.

It is interesting to note that the current status of fiber OPAs is such that it would probably be possible to use them today to handle the capacity of long-haul fiber optic systems of a decade ago. However the capacity of these systems has increased in a Moore's-law-type fashion, and so the requirements for OPAs to be competitive with today's leading-edge systems have become very challenging. The recently-demonstrated ability of OPAs to amplify high-order modulation formats with little penalty is encouraging. However, other developments are more problematic. For example, coherent detection (CD) and digital signal processing (DSP) are now becoming major attributes of commercial systems. One reason for this is that this combination can be used for combating nonlinear effects in the transmission lines, and greatly reduce the need for optical dispersion compensation in the network. This poses a challenge for the insertion of PSAs in such networks, because the currently-envisioned architecture for PSA-based systems assumes perfect optical dispersion compensation before every PSA. Other emerging trends, such as multiple-core or multimode amplification, and polarization-division multiplexing (PDM) could pose additional challenges.

Hence we see that research work on fiber OPAs needs to steadily progress in order to have a chance of catching up with the ever-moving target presented by the requirements of state-of-the-art fiber optical communication systems. The recent demonstration of transmission of an 8-channel WDM 32-GBd PDM QPSK signal over 6,000-km by periodic phase conjugation with phase-insensitive OPAs is an excellent demonstration of the potential of fiber OPAs in communication systems [86].
